# Rational Design and Precise Synthesis of Single‐Atom Alloy Catalysts for the Selective Hydrogenation of Nitroarenes

**DOI:** 10.1002/advs.202304908

**Published:** 2024-04-10

**Authors:** Haisong Feng, Wei Liu, Lei Wang, Enze Xu, Donghui Pang, Zhen Ren, Si Wang, Shiquan Zhao, Yuan Deng, Tianyong Liu, Yusen Yang, Xin Zhang, Feng Li, Min Wei

**Affiliations:** ^1^ State Key Laboratory of Chemical Resource Engineering Beijing Advanced Innovation Center for Soft Matter Science and Engineering Beijing University of Chemical Technology Beijing 100029 P. R. China; ^2^ Quzhou Institute for Innovation in Resource Chemical Engineering Quzhou 324000 P. R. China

**Keywords:** catalysts designed, density functional theory, host–guest metal interactions, selective hydrogenation, single‐atom alloy catalysts

## Abstract

Single‐atom alloys (SAAs) have gained increasing prominence in the field of selective hydrogenation reactions due to their uniform distribution of active sites and the unique host‐guest metal interactions. Herein, 15 SAAs are constructed to comprehensively elucidate the relationship between host‐guest metal interaction and catalytic performance in the selective hydrogenation of 4‐nitrostyrene (4‐NS) by density functional theory (DFT) calculations. The results demonstrate that the SAAs with strong host‐guest metal interactions exhibit a preference for N─O bond cleavage, and the reaction energy barrier of the hydrogenation process is primarily influenced by the host metal. Among them, Ir_1_Ni SAA stands out as the prime catalyst candidate, showcasing exceptional activity and selectivity. Furthermore, the Ir_1_Ni SAA is subsequently prepared through precise synthesis techniques and evaluated in the selective hydrogenation of 4‐NS to 4‐aminostyrene (4‐AS). As anticipated, the Ir_1_Ni SAA demonstrates extraordinary catalytic performance (yield > 96%). In situ FT‐IR experiments and DFT calculations further confirmed that the unique host‐guest metal interaction at the Ir‐Ni interface site of Ir_1_Ni SAA endows it with excellent 4‐NS selective hydrogenation ability. This work provides valuable insights into enhancing the performance of SAAs catalysts in selective hydrogenation reactions by modulating the host‐guest metal interactions.

## Graphical abstract

Through the exploration of host–guest interactions of single‐atom alloys (SAA) via DFT calculations, a high‐performance Ir_1_Ni SAA catalyst is designed for the selective hydrogenation of 4‐nitrostyrene. Subsequently, the Ir_1_Ni SAA is meticulously prepared using precise synthesis techniques and assessed in the selective hydrogenation of 4‐nitrostyrene to 4‐aminostyrene. As anticipated, the Ir_1_Ni SAA exhibits exceptional catalytic performance, with a yield exceeding 96%.

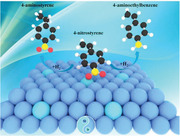

## 1 Introduction

Selectivity hydrogenation is a fundamental and crucial topic in heterogeneous catalysis; therefore, achieving both high selectivity and conversion is of utmost importance in designing heterogeneous catalysts.^[^ ^1^, ^2^, ^3^, ^4^, ^5^ ^]^ Among various hydrogenation reactions, the selective hydrogenation of aromatic nitro compounds holds significant importance as it enables the production of functionalized anilines, which serve as crucial intermediates in the synthesis of pharmaceuticals, dyes, and other fine chemicals.^[^ ^6^, ^7^, ^8^, ^9^, ^10^ ^]^ Pt‐group metals have emerged as promising heterogenous catalysts for nitro reduction due to their high activity.^[^ ^11^, ^12^, ^13^ ^]^ However, aromatic nitro compounds contain other reducible groups, such as C═C, C═O, C≡C, and C≡N. The reduction of nitro groups by traditional Pt‐group metal catalysts often also leads to the reduction of the undesired unsaturated groups because these reactions are kinetically and thermodynamically favorable than nitro group reduction.^[^ ^14^, ^15^, ^16^, ^17^, ^18^ ^]^ Therefore, rational design of catalysts that can precisely control the hydrogenation of nitro groups remains a significant challenge.

An ideal catalyst that promotes the preferential activation of the nitro group requires modification of Pt‐group metals with other metals to facilitate this process. One of the effective strategies is to introduce Pt‐group metals into less active component, which can combine the strengths of individual components and overcome their weaknesses.^[^ ^19^ ^]^ For instance, catalysts such as Pd_13_Pb_9_,^[^ ^12^ ^]^ RhCd,^[^ ^20^ ^]^ RhCo,^[^ ^21^ ^]^ PtZn^[^ ^22^ ^]^ and PtSn^[^ ^23^ ^]^ not only demonstrated high activity in selective hydrogenation of nitrostrene, but also exhibited high chemo‐selectivity toward aminostyrene. Upon precise design, Pt‐group metal atoms might be isolated into single atom on less active metal surfaces. The unique alloy mode significantly alters the electronic structure of isolated metal atoms, resulting in distinct activity, selectivity, and stability compared to conventional metal catalysts.^[^ ^24^, ^25^, ^26^ ^]^ Additionally, the formation of single atom alloy (SAA) can modify the adsorption configuration of reaction species and the reaction pathway, offering the potential to catalyze the reaction in the desired direction.^[^ ^27^, ^28^, ^29^, ^30^, ^31^, ^32^, ^33^ ^]^ However, researches devoted to improve the catalytic selectivity of Pt‐group metal for selective hydrogenation of nitroarenes by modulating Pt‐group metals and host metals atoms and forming SAA strategy are still scarce.

In this work, three non‐noble metals (Co, Ni, and Cu) with excellent hydrogenation capabilities were selected as the host metal, and five Pt‐group metals (Ru, Rh Pd, Ir, and Pt) were selected as single‐atom metals. A total of 15 SAAs, denoted as M_1_M_host_ (M_1_ = Ru, Rh, Pd, Ir, and Pt, M_host_ = Co, Ni, and Cu), were constructed to evaluate catalytic performance and reveal the relationship between host and guest metal interactions in selective hydrogenation of 4‐nitrostyrene (4‐NS) by density functional theory (DFT). Considering the activation of N−O bond, C_8_H_7_NOH hydrogenation, and vinyl hydrogenation, we obtained two SAAs, Ru_1_Ni, and Ir_1_Ni, with high potential for catalytic selective hydrogenation of 4‐NS. Subsequently, the Ir_1_Ni SAA, identified as the best candidate catalyst, was further prepared by precise synthesis technique, confirmed the formation of Ir_1_Ni SAA by detailed characterizations, and evaluated in the selective hydrogenation of 4‐nitrostyrene to 4‐aminostyrene (4‐AS). In accordance with expectation, the Ir_1_Ni SAA exhibits extraordinary catalytic performance in the conversion of 4‐NS to 4‐AS (selectivity > 98%). This work provides valuable guidance for exploring the host–guest interaction in SAA and developing effective catalysts for selective hydrogenation reactions.

## 2 Results and Discussion

### 2.1 Investigation on the Suitability Between SAA Models and 4‐NS

**Figure 1:**
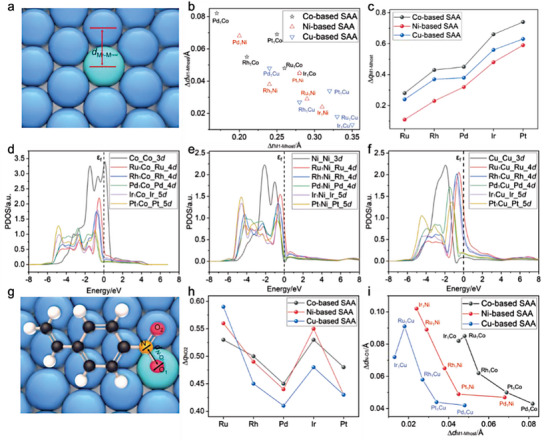
a) The structure of M_1_M_host_ SAAs (M_1_ = Ru, Rh, Pd, Ir, and Pt, M_host_ = Co, Ni and Cu). b) Relationship between the variation of metal bond length and the difference of the radius between host and guest metals (∆*d*_M1─Mhost_ = *d*_M1−Mhost_ − *d*_Mhost−Mhost_). c) The number of electron transfer form host metal to guest metal. Projected density of states (PDOS) for d) M_1_Co, e) M_1_Ni, and f) M_1_Cu SAAs. g) The most stable adsorption configuration of 4‐NS over the SAA surfaces. h) The electrons gained by the nitro group on surfaces of SAAs. i) Relationship between Δ*d*_N‐O1_ and Δ*d*_M1‐Mhost_ on different SAA surfaces.

To illustrate the synergistic effect and the suitability between host and guest metals, the geometric structure and electronic structure of 15 SAAs were explored. The results show the bond lengths of host and guest metals (M_1_−M_host_) on the SAA surface are affected by the atomic radius. A larger difference in radii between the host and guest metals resulted in shorter M_1_−M_host_ bond length (**Figure** **1**a,b). This can be attributed to the varying strengths of interaction between the host and guest metals, with stronger interactions leading to shorter bond lengths. Electronic structure analysis reveals that the electrons are transferred from the host metal to single atom, resulting in the single atom carrying negative charges$\;M_{1}^{\delta^{-}}$. As the number of protons in the single atom metal increased, the amount of negative charge also increased (Figure 1c). Additionally, the projected density of states (PDOS) of 15 SAAs was calculated, as shown in Figures S1−S5 (Supporting Information) and Figure 1d−f. The *d*‐band width of the single atom in SAAs was observed to be narrow and maintained good symmetry compared with pure bulk noble metals, which could be responsible for enhanced catalytic activity exhibited by these SAAs in previous research.^[^ ^25^, ^34^ ^]^ Furthermore, the PDOS peaks of Ru and Ir single atoms were found to be closer to the Fermi level. This may result in stronger adsorption and activation capacities of Ru_1_M_host_ and Ir_1_M_host_ SAA for the substrate compared to Rh_1_M_host_, Pd_1_M_host,_ and Pt_1_M_host_ SAAs.

The adsorption configuration of the substrate with the catalyst surface is known to play an important role in the heterogeneous catalysis. Therefore, three parallel and two vertical adsorption configurations of nitro group were considered on the surface of SAAs around the single‐atom active center (Figure S6, Supporting Information). For two vertical adsorption modes, the 4‐NS molecule was placed vertically on the surface, with two oxygen atoms of the nitro group are bonded to adjacent M_host_−M_host_ (named V_O−Mhost_) or M_1_−M_host_ (named V_O−M1_), respectively. In the case of the three parallel adsorption modes, the 4‐NS molecule was oriented parallel to the surface, and we considered three configurations: 1) where an oxygen atom on the nitro group was adsorbed on the M_1_ site (referred to as P_O−M1_), 2) where nitrogen atom was adsorbed on the M_1_ site (referred to as P_N−M1_), 3) where all the nitrogen and oxygen atoms were adsorbed on the host metal site (referred to as P_O−Mhost_). As an example, the five adsorption configurations of 4‐NS over Ru_1_Co SAA were discussed in detail. The adsorption structure and binding energy of 4‐NS on Ru_1_Co(111) are shown in Figure S7 (Supporting Information). By comparing the vertical and parallel adsorption, it was found that the phenyl and vinyl group could adsorb on the surface via the formation of C─Co bonds in parallel adsorption mode. The binding energy of parallel adsorption is about 2 eV higher than that vertical adsorption (*E*_b_ = 1.32, 1.56, 3.40, 3.10, and 3.30 eV for V_O−Co_, V_O−Ru_, P_O−Ru_, P_N−Ru,_ and P_O−Co_ over Ru_1_Co SAA, respectively). This indicates that the parallel adsorption modes are more thermodynamically stable than vertical adsorption modes. Furthermore, among the parallel adsorption modes, the P_O−Ru_ mode is 0.10 eV higher than the other two modes, indicating its higher stability (Figure 1g). The calculated results of the adsorption configurations on Ru_1_Ni(111) and Ru_1_Cu(111) are consistent with the Ru_1_Co(111) (Figures S8 and S9, Supporting Information). In addition, we also considered the adsorption of vinyl groups on single‐atom sites (named P_C‐M1_), and the results are shown in Figure S10 (Supporting Information). The findings reveal that, with the exception of Ru_1_Cu, Rh_1_Cu, and Ir_1_Cu, the binding energy associated with the P_O‐M1_ adsorption mode on other SAA surfaces is higher than that of P_C‐M1_. Notably, the disparity in binding energy between the two adsorption modes exceeds a substantial margin of 0.15 eV, underscoring the remarkable superiority of the P_O‐M1_ adsorption mode over the P_C‐M1_ adsorption mode. Despite the higher binding energy of P_C‐M1_ compared to P_O‐M1_ on the surfaces of Ru_1_Cu and Ir_1_Cu, we have chosen to investigate the P_O‐M1_ adsorption mode in our next step to explore the reaction mechanism. This decision is driven by our keen interest in understanding the influence of host–guest metal interactions on nitro reduction. Therefore, the optimal adsorption configuration for further exploration was determined to be the adsorption of an O atom of the nitro group at the single atom top site.

The analysis of charge density difference (CDD) for Ru_1_Co, Ru_1_Ni, and Ru_1_Cu (Figures S11−S13, Supporting Information) reveals that upon the adsorption of 4‐NS molecules onto the surface of the SAA, a substantial transfer of electrons transpires between 4‐NS and the surface, engendering a robust interaction. This phenomenon suggests the potential presence of a covalent bond interaction, further enhancing the strength of interplay between the nitro group and the surface of the SAAs. Bader charge of P_O‐Ru_ adsorption configurations are also calculated (Figure 1h), it shows that there is a significant electron transfer between 4‐NS and SAAs. Moreover, compared to the benzene ring and vinyl groups in 4‐NS, the nitro groups exhibit a more significant enhancement in electron density due to the electron transfer from M_1_−M_host_ interface, suggesting the specific activation of the N−O bond in nitro group. Interestingly, even though the Ru atom receives the least number of electrons in SAA surfaces (Figure 1c), it transfers the greatest number of electrons transferred to nitro groups (Figure 1h). This implies that the number of electrons in the SAA itself may have minimal effect on the activation of 4‐NS. Additionally, the Ir single atom also transfers a large number of electrons to the nitro group, indicating that Ru_1_M_host_ and Ir_1_M_host_ SAAs may have strong activating ability for reactant molecules. To further explore the relationship between structure of SAAs and adsorption activation of reactant molecule, the binding energy of 4‐NS on the surface of 15 SAAs and the *d*‐band center of surface single atom were analyzed (**Table** **1**). It demonstrates that a higher *d*‐band center value corresponds to a higher binding energy for adsorbent molecules. The binding energy of 4‐NS will be greatly changed by simply changing the single atom (e.g., the binding energy of 4‐NS on Ru_1_Co(111) (*E*_ads_ = 3.40 eV) is 0.33 eV higher than the binding energy of 4‐NS on Pd_1_Co(111) (*E*_ads_ = 3.07 eV)).This highlights the significant influence of the compatibility between the single atom and the host metal on the adsorption activation of 4‐NS.

**Table 1:** The binding energies of 4‐NS and the *d*‐band center of single atom on M_1_M_host_(111).

Entry	Surface	*d*‐band Center Value [eV]	Binding Energy [eV]
1	Co(111)	−1.41	3.36
2	Ru_1_Co(111)	−1.53	3.40
3	Rh_1_Co(111)	−1.95	3.22
4	Pd_1_Co(111)	−2.50	3.07
5	Ir_1_Co(111)	−2.07	3.18
6	Pt_1_Co(111)	−2.68	3.00
7	Ni(111)	−1.73	3.14
8	Ru_1_Ni(111)	−1.41	3.29
9	Rh_1_Ni(111)	−1.61	3.16
10	Pd_1_Ni(111)	−1.96	2.89
11	Ir_1_Ni(111)	−1.93	2.97
12	Pt_1_Ni(111)	−2.33	2.98
13	Cu(111)	−2.04	1.80
14	Ru_1_Cu(111)	−0.80	2.07
15	Rh_1_Cu(111)	−1.67	1.89
16	Pd_1_Cu(111)	−2.04	1.70
17	Ir_1_Cu(111)	−0.88	1.88
18	Pt_1_Cu(111)	−1.84	1.66

The geometry of adsorbed 4‐NS was also investigated by the change of the N−O_1_ bond length. Compared to 4‐NS in gaseous state, the N−O_1_ bond length increased by 0.042−0.102 Å (Δ*d*_N‐O1_) when 4‐NS is adsorbed on M_1_M_host_(111) (Figure 1i; Table S1, Supporting Information). It is observed that stronger host–guest metal interactions tend to promote the growth of N−O_1_ bond. Therefore, Ru_1_M_host_ and Ir_1_M_host_ SAAs appear to be good candidate catalysts for the reduction of 4‐NS.

### 2.2 Reduction of 4‐NS over SAA Surfaces

**Figure 2:**
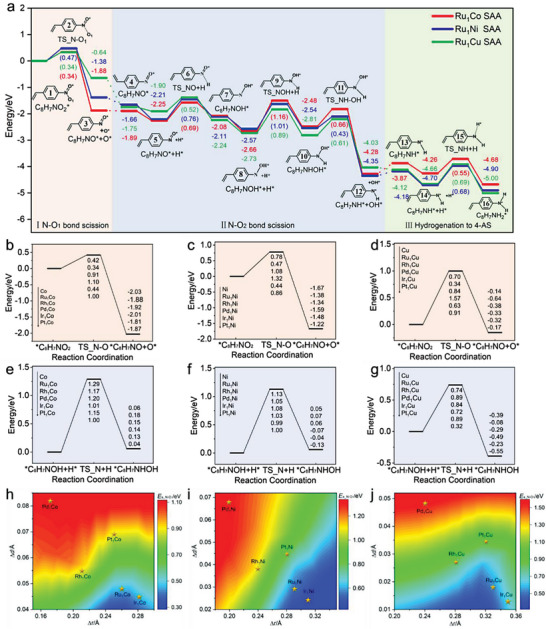
a) The energy profiles of 4‐NS reduction over Ru_1_Co(111), Ru_1_Ni(111) and Ru_1_Cu(111), the results on Ru_1_Ni SAA calculated by our previous work^[^ ^35^ ^]^ (The inclusion of dashed connections in the figure signifies that certain less crucial elementary reaction steps were not explored in our study). Potential energy profiles of the N−O_1_ bond breaking step on the surface of b) M_1_Co SAAs, c) M_1_Ni SAAs and d) M_1_Cu SAAs. Potential energy profiles of the C_8_H_7_NOH hydrogenation step on the surface of e) M_1_Co SAAs, f) M_1_Ni SAAs and g) M_1_Cu SAAs. The energy barrier maps for N−O_1_ bond scission as a function of the variation of metal bond length and the difference of the radius between host and guest metals on the surface of h) M_1_Co SAAs, i) M_1_Ni SAAs and j) M_1_Cu SAAs.

In order to understand the influence of the synergistic effect between the single atom and the host metal on the reduction of 4‐NS, the reaction pathways for the reduction of 4‐NS over Ru_1_M_host_(111) were investigated (the results on Ru_1_Ni SAA calculated by our previous work).^[^ ^35^ ^]^ The conversion of 4‐NS to 4‐AS involves three main chemical processes: the N−O_1_ bond scission, N−O_2_ bond scission and hydrogenation to 4‐AS. The detailed elementary steps, related structures and the corresponding potential energy profiles are summarized in **Figure** **2**a and Figures S14–S16 (Supporting Information).

The cleavage of the N−O bond is the initial step in the hydrogenation of C_8_H_7_NO_2_* to C_8_H_7_NH_2_*, and its efficiency is a crucial indicator for evaluating catalyst performance. Moreover, it determines whether the nitro group can be preferentially reduced to amino group.^[^ ^22^, ^35^ ^]^ There are two possible routes for the N−O_1_ bond scission: i) direct breakage of N−O bond and ii) H‐ assisted dissociation. Our previous experimental results have shown that 4‐NS undergoes direct breakage of the N−O bond over Ru_1_Ni(111).^[^ ^35^ ^]^ In addition, our calculated results also show that the H‐assisted intermediate C_8_H_7_NOH cannot stably exist on the catalyst surface, including Co(111), Ni(111) Cu(111) and M_1_M_host_(111) (M_1_ = Co, Ni and Cu, M_host_ = Ru, Rh, Pd, Ir, and Pt). Therefore, direct deoxygenation is considered to be the preferred path for the N−O_1_ bond scission on M_1_M_host_(111). As shown in Figure 2a, the N−O_1_ on the Ru‐M_host_ interface sites was breakage directly, and the energy barrier is about 0.40 eV for the direct N−O_1_ bond dissociation of 4‐NS over Ru_1_M_host_, which is exothermic, indicating its thermodynamic favorability.

After the first N−O bond breaking, the resulting C_8_H_7_NO* undergoes two steps hydrogenation to generate C_8_H_7_NHOH*. In the first place, the active hydrogen atoms situated at Ru−M_host_ hollow sites initiate an attack on the oxygen atom, giving rise to C_8_H_7_NOH*, which requires moderate energy barriers about 0.70 eV. In the next place, the N atom in C_8_H_7_NOH* is hydrogenation to produce C_8_H_7_NHOH* after overcoming energy barriers of more than 0.89 eV, and it is the rate‐determining step for the whole hydrogenation process because its energy barrier is higher than those of all the hydrogenation steps. Subsequently, the N−OH bond within C_8_H_7_NHOH* undergoes scission at Ru−M_host_ interface sites, necessitating lower energy barriers (∼0.60 eV) and resulting in an exothermic release of over 1.00 eV. It indicates that this step is also facile. Finally, the formed C_8_H_7_NH* can be further hydrogenated to produce 4‐AS with energy barriers of about 0.70 eV.

In summary, the steps of N−O_1_ bond breakage and C_8_H_7_NOH* hydrogenation are of vital significant to selective hydrogenation of 4‐NS. The former is the selectivity‐determining step, while the latter is the rate‐determining step for the overall 4‐NS selective hydrogenation. Therefore, we will discuss the two key steps in 4‐NS reduction over all the M_1_M_host_(111) to reveal the relationship between host–guest synergy and catalytic performance (the potential energy profiles are shown in Figure 2b–g), and screen out a class of catalysts that exhibit excellent activity and selectivity for 4‐NS reduction.

The N−O_1_ bond scission ability of SAA can be adjusted by changing the host and guest metals. Figure 2b–d shows the energy barriers of N−O_1_ bond scission, which are 0.42, 0.78, and 0.70 eV over the Co(111), Ni(111) and Cu(111), respectively. Doping single‐atom Ru and Ir on the surface of the host metal significantly reduces the N−O_1_ bond scission energy barrier (the energy barriers in the range of 0.34−0.63 eV). This indicates that the incorporation of Ru and Ir atoms promotes the breaking of the N−O_1_ bond. However, there are high energy barriers of N−O_1_ bond scission on Rh_1_M_host_, Pd_1_M_host_ and Pt_1_M_host_ SAAs (the energy barriers in the range of 0.84−1.57 eV), indicating that single‐atom Rh, Pd, and Pt doping on the surface of the host metal M_host_ are not conducive to the N−O_1_ bond breaking. To investigate the effect of the interaction between host and guest metals in SAAs on the activation ability of N−O_1_ bond, the relationship between the SAA geometry of and the energy barrier for N−O bond scission was explored. As shown in Figure 2h–j, stronger host–guest metal interactions are more conducive to the breaking of N−O_1_ bonds. This may be due to the strong interaction that leads to a better match between the bond lengths of the host and guest metals and the N─O bond lengths (1.224 Å in gas phase 4‐NS molecule), resulting in better activation of the N−O_1_ bond.

The potential energy profile for the C_8_H_7_NOH* hydrogenation process on M_host_(111) and M_1_/M_host_(111) is depicted in Figure 2e–g. Although the N−O_1_ bond is easily broken over the Co(111), the C_8_H_7_NOH* hydrogenation is not expected to be facilitated due to the high reaction barrier (1.29 eV). For Ni(111) and Cu(111), the hydrogenation energy barriers of C_8_H_7_NOH* are 1.13 and 0.74 eV, respectively. It indicates that C_8_H_7_NOH* hydrogenation is more favorable on Cu(111) compared to Co(111) and Ni(111). For M_1_Co SAAs, it can be observed that the energy barrier for C_8_H_7_NOH* hydrogenation decreases with the incorporation of single atom metals, falling within the regions of 1.00−1.20 eV. Similarly, for M_1_Ni SAA catalysts, low energy barriers about 1.00 eV of C_8_H_7_NOH* hydrogenation are obtained. It is worth noting that the hydrogenation energy barriers are relatively similar over M_1_Co or M_1_Ni SAA surfaces, suggesting that they may exhibit comparable capabilities for C_8_H_7_NOH* hydrogenation. In the case of M_1_Cu SAA, only the incorporation of Pt metals can enhance the hydrogenation performance with an energy barrier of 0.32 eV. Other Cu‐based SAA surfaces have similar C_8_H_7_NOH* hydrogenation capabilities (the hydrogenation energy barrier is about 0.80 eV), far superior to M_1_Co and N_1_Ni SAAs. This suggests that the energy barrier for C_8_H_7_NOH* hydrogenation may be influenced by the choice of host metal.

### 2.3 Performance Prediction of SAA Catalysts

Vinyl hydrogenation is a side reaction of 4‐NS selective hydrogenation. Therefore, the competition between nitro group reduction and vinyl reduction must be considered. Hence, the first hydrogenation steps of vinyl were investigated. For nitro group adsorption on single atom sites, the vinyl is far away from the single atom, and the hydrogenation of the vinyl may not be affected by single atom. To verify our hypothesis, the vinyl hydrogenation process over M_host_(111) and Ru_1_M_host_(111) were investigated (Figures S17–S19, Supporting Information). The results showed that the difference in hydrogenation energy barrier is less than 0.05 eV, indicating that the incorporation of single atoms has little effect on the energy barrier of vinyl hydrogenation. Therefore, the hydrogenation energy barrier of vinyl over pure Co(111), Ni(111) and Cu(111) were calculated to represent the hydrogenation energy barrier of vinyl over M_1_Co(111), M_1_Ni(111) and M_1_Cu(111). The energy barriers of vinyl hydrogenation are 0.38, 0.69, and 0.40 eV over Co(111), Ni(111) and Cu(111), respectively. This observation suggests that the reduction of vinyl groups is more favorable than the reduction of nitro groups on the M_1_Cu(111) surface.

**Figure 3:**
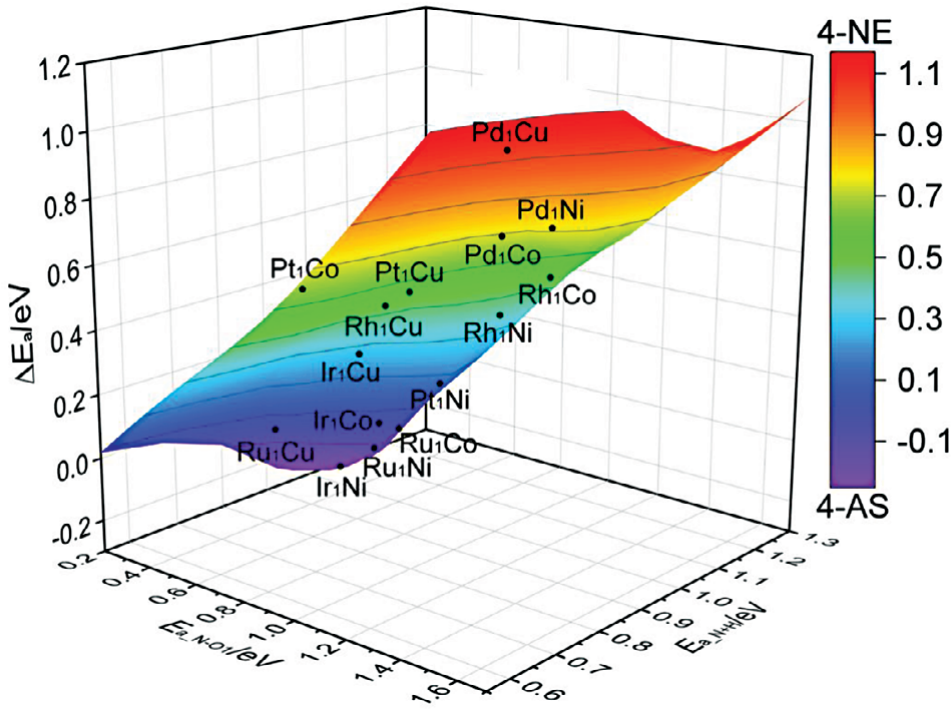
Selectivity of 4‐NS hydrogenation as a function of the energy barrier of N−O_1_ scission and the energy barrier of C_8_H_7_NOH hydrogenation over M_1_M_host_ SAAs (M1 = Ru, Rh, Pd, Ir, and Pt, M_host_ = Co, Ni, and Cu).

The selectivity of SAA catalysts for the selective hydrogenation of 4‐NS can be evaluated by comparing the energy barrier difference between N−O_1_ bond scission and vinyl hydrogenation. A more negative energy barrier difference indicates higher selectivity toward 4‐AS.^[^ ^22^, ^35^ ^]^ And the nitro‐reducing activity of SAA catalysts can be assessed based on the energy barrier of C_8_H_7_NOH hydrogenation, with lower values indicating higher activity. As shown in **Figure** **3**, selectivity appears to be the main factor affecting the catalytic performance of SAAs. Among the catalysts studied, only Ru_1_Co, Ru_1_Ni, Ir_1_Ni, and Ru_1_Cu catalysts exhibit a negative energy barrier difference for hydrogenation. However, on the Ru_1_Co and Ru_1_Cu surfaces, the energy barrier differences between nitro activation and vinyl hydrogenation are too small to distinguish the selectivity of nitro reduction from vinyl reduction. Therefore, it can be speculated that Ru_1_Ni and Ir_1_Ni SAAs have the potential to achieve decent catalytic performance for the selective hydrogenation of 4‐NS. Thereinto, the catalytic performance of Ir_1_Ni SAA is expected to be the best.

### 2.4 Ni‐Based SAA Preparation and Reaction Evaluation

**Figure 4:**
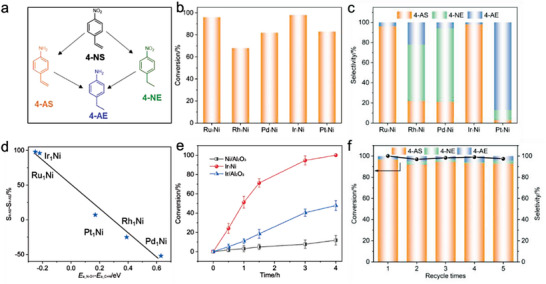
a) Reaction network of the hydrogenation of 4‐nitrostyrene. b) Catalytic conversion of 4‐NS hydrogenation and c) product distribution on different SAA catalysts. d) The relationship between the predicted selectivity of DFT calculation and the experimental results (S_4‐AS_ and S_4‐NE_ represent the selectivity of 4‐AS and 4‐NE, respectively. *E*_a_N−O1_ and *E*_a_C+H_ are the N−O_1_ bond breaking energy barrier and the vinyl first‐step hydrogenation energy barrier, respectively). e) Catalytic conversion of 4‐nitrostyrene hydrogenation over pristine Ni, pristine Ir, and Ir_1_Ni SAA samples. f) Reusability tests of Ir_1_Ni catalyst within five successive catalytic cycles. (Reaction condition: 1 mmol of 4‐NS; 8 mL of ethanol; 0.03 g of catalyst; 1 MPa of H_2_, 50 °C, 4 h).

To further access the performance of the screened SAA catalysts, as well as the DFT results, a set of M_1_Ni/Al_2_O_3_ (M_1_ = Ru, Rh, Pd, Ir, and Pt) SAA was precisely synthesized using a galvanic replacement method to deposit single atom metals onto the surface of Ni nanoparticles,^[^ ^35^ ^]^ and then the relative catalytic evaluations were performed. Briefly, Ni nanoparticles supported on amorphous Al_2_O_3_ substrate were prepared based on structural topotactic transformation of layered double hydroxides (LDHs) precursors. Subsequently, a set of M_1_Ni/Al_2_O_3_ bimetallic samples supported by different noble metals was accurately synthesized by depositing noble metal atoms on the surface of Ni nanoparticles using the current displacement method (the loading of noble metals is ≈0.6%). The catalytic performance of various M_1_Ni/Al_2_O_3_ catalysts for the chemoselective hydrogenation of 4‐NS to 4‐AS was investigated, and the results are presented in **Figure** **4**. Under the conditions of 1 MPa H_2_ at 50 °C, the Ir_1_Ni/Al_2_O_3_ and Ru_1_Ni/Al_2_O_3_ catalysts exhibit desirable conversion (>96%) and selectivity (>96% for 4‐AS) (Figure 4a–c), indicating they are exclusively chemoselective for nitro group rather than ethenyl, which accords well with the result that the N─O_1_ bond breaking energy barrier is lower than that of vinyl hydrogenation obtained by DFT calculations. The selectivity of 4‐AS for Rh_1_Ni, Pd_1_Ni and Pt_1_Ni/Al_2_O_3_ samples, by contrast, dropped to 22%, 21% and 3%, respectively (Figure 4c). In the case of Pt_1_Ni/Al_2_O_3_, both nitro groups and vinyl groups were reduced simultaneously, resulting in 87% selectivity for 4‐aminoethylbenzene (4‐AE). For Rh_1_Ni/Al_2_O_3_ and Pd_1_Ni/Al_2_O_3_ samples, 56% and 73% of 4‐nitroethylbenzene (4‐NE) was formed, respectively. These results also align with the DFT calculations, where the energy barrier difference between N−O_1_ bond scission and vinyl hydrogenation were significantly higher for Rh_1_Ni/Al_2_O_3_ and Pd_1_Ni/Al_2_O_3_ catalysts (0.39 and 0.63 eV, respectively). The comparison of the barrier difference between N−O_1_ scission and vinyl first‐step hydrogenation calculated by DFT with the selectivity obtained experimentally (Figure 4d) demonstrated that a more negative energy barrier difference corresponds to a more favorable selectivity for 4‐AS. This confirms the reliability of using Δ*E*_a_ calculated by DFT as the selective screening criterion. Both experimental and computational results highlight the superior catalytic performance of the Ir_1_Ni catalyst. Furthermore, the catalytic performance of pristine Ni and Ir catalysts were also explored toward chemoselective hydrogenation of 4‐NS to 4‐AS. It worth mentioning that the pristine Ni and Ir catalysts exhibit limited reactivity, with conversions of 11.8% and 47.8% after 4 h, respectively, and poor selectivity toward 4‐AS (55.2% and 21.8%, respectively, Figure 4e; Figure S20, Supporting Information), demonstrating the unique catalytic properties of SAA. The stability of the Ir_1_Ni/Al_2_O_3_ sample was further evaluated, and the activity and selectivity can still be maintained above 96% within five successive recycles (Figure 4f), which demonstrates its practical applicability.

### 2.5 Structural Characterizations of Ir_1_Ni SAA

**Figure 5:**
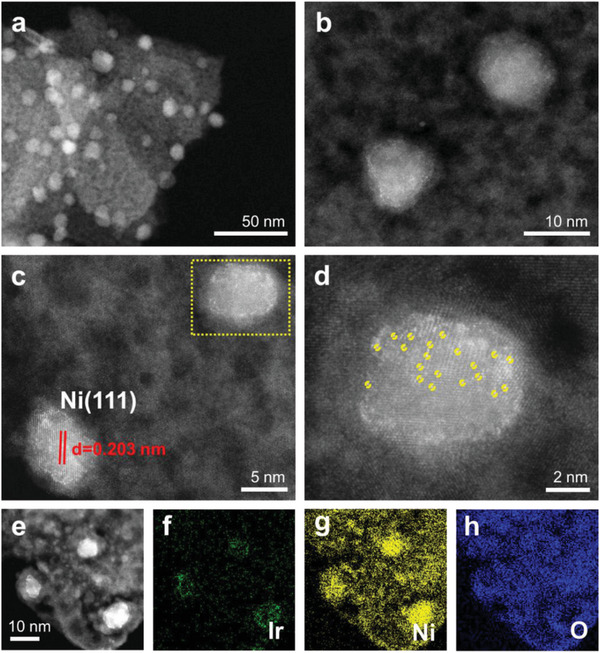
a–e) High resolution AC‐HAADF‐STEM image of Ir_1_Ni SAA sample. Element EDS mapping images of f) Ir, g) Ni and h) O, respectively.

The structure of Ir_1_Ni/Al_2_O_3_ SAA was confirmed through various characterization techniques, such as X‐ray diffraction (XRD), aberration‐corrected high angle annular dark‐field scanning transmission electron microscopy (AC‐HAADF‐STEM) images and energy‐dispersive spectroscopy (EDS) elemental mapping. Scanning eletron microscope (SEM) images (Figure S21, Supporting Information) clearly reveal that the prepared samples showed a regular nanoflower‐like multi‐level structure. The XRD patterns of Ir_1_Ni nanocrystals (Figure S22, Supporting Information) show the peaks assigned to (111), (200) and (220) of a typical Ni (JCPDS 004–0850) phase, which are similar position to the Ni/Al_2_O_3_ sample. Notably, no metallic or oxidized Ir reflections were observed for the Ir_1_Ni sample, implying a high dispersion of Ir species. Furthermore, the XRD pattern (Figure S23, Supporting Information) of the used Ir_1_Ni SAA catalyst after five cycles do not show any significant changes. AC‐HAADF‐STEM images (**Figure** **5**a–e) demonstrate the presence of individual brighter Ir atoms (highlighted by yellow circles) in Ni nanoparticles, without observation of Ir clusters or nanoparticles. The lattice spacing of Ir_1_Ni is 0.203 nm, which is in good consistence with the lattice spacing of Ni(111), indicating the replacement of surface Ni atoms by isolated Ir atoms and atomic dispersion of Ir atoms in Ni nanoparticles. Further EDS elemental mapping (Figure 5f–h) analysis exhibited a highly uniform dispersion of Ir on Ni nanoparticles rather than Al_2_O_3_ substrate. Moreover, no significant change was observed after five cycles (Figure S24, Supporting Information).

**Figure 6:**
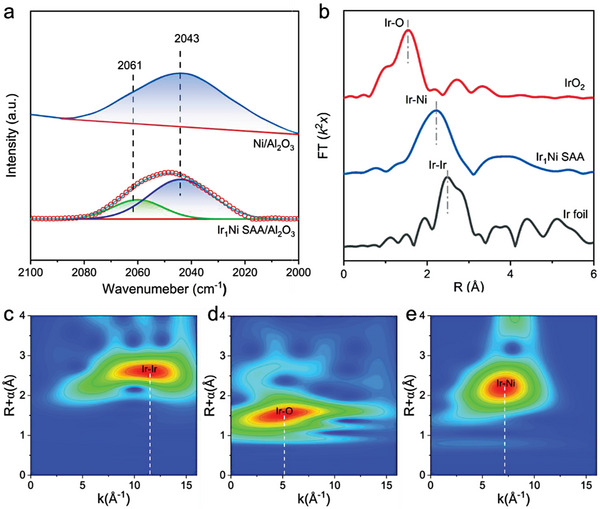
a) In situ CO‐DRIFTS spectra of Ni/Al_2_O_3_ sample and the enlarged and Gaussian fitting spectra with a fixed peak position and FWHM of Ir_1_Ni SAA/Al_2_O_3_ sample. b) EXAFS Fourier‐transform spectra of Ir_1_Ni SAA sample. *k* ^2^‐weighted wavelet transforms for the Ir K‐edge XAFS signals of c) Ir foil, d) IrO_2_, and e) Ir_1_Ni SAA sample based on Morlet wavelets. “a. u.” denotes arbitrary units.

In situ CO–DRIFTS spectra of CO adsorption were performed to further probe the atomic geometry configuration of Ir atoms on the Ir_1_Ni SAA sample. As show in **Figure** **6**a, CO adsorption on Ni/Al_2_O_3_ sample produces a dominant peak at 2043 cm^−1^, which is ascribed to the linear CO on Ni site. In comparison, another prominent peak centered at 2061 cm^−1^ is observed on the Ir_1_Ni SAA, which can be attributed to the symmetric stretching vibration peak of the Ir(CO)_2_ species on the single Ir atom.^[^ ^36^, ^37^, ^38^ ^]^ Moreover, no other distinct vibrational peaks for the linear adsorption of CO on Ir nanoparticles were found in the range of 2030–2090 cm^−1^. This observation suggests that the Ir atoms on the Ir_1_Ni SAA are highly dispersed, which accords well with the AC–HAADF–STEM results. To verify the electronic structure and coordination structure of Ir_1_Ni SAA, X‐ray photoelectron spectroscopy (XPS) and extend X‐ray absorption fine spectrum (EXAFS) spectra were performed. As shown in Figure S25 (Supporting Information), the XPS analysis unveiled a discernible shift of the Ir 4*d* peak toward lower binding energy in the Ir_1_Ni/Al_2_O_3_ sample, in contrast to the Ir/Al_2_O_3_ sample. The Ni 2*p* peak of Ir_1_Ni/Al_2_O_3_ is shifted to higher binding energy than metallic Ni/Al_2_O_3_. Moreover, the white line intensity of Ir Kedge XANES spectra show a slight shrink from Ir/Al_2_O_3_ to Ir_1_Ni SAA samples (Figure S26, Supporting Information). These phenomena indicate electron transfer from Ni atoms to Ir atoms in Ir_1_Ni/Al_2_O_3_ sample, which is consistent with Bader charges analysis results obtained from DFT calculations. The detailed coordination structure of Ir was investigate by the Fourier transforms of the EXAFS in the R space (Figure 6b). There is a sharp peak located at ∼2.2 Å, which is in the region between Ir─O shell (1.5 Å) and Ir─Ir shell (2.5 Å) and can be assigned to the Ir─Ni coordination. The coordination environment of Ir species is further study by the *k* ^2^‐weighted wavelet transform (WT) analysis of the Ir K‐edge X‐ray absorption fine structure spectroscopy (XAFS) signals. Additionally, as illustrated by the WT contour plots of Ir foil and IrO_2_ (Figure 6c–e), the intensity maxima at 11.7 and 5.1 Å^−1^ are attributed to the Ir─Ir and Ir─O contributions, respectively. For the WT contour plot of Ir_1_Ni SAA sample, the absence of peak at 11.7 and 5.1 Å^−1^ excludes the central Ir bonds to Ir atom and O atom, meanwhile, a new intensity crest was observed at near 7.3 Å^−1^, which is assigned to the Ir─Ni contribution, further indicating that the formation of the formation of Ir_1_Ni SAA catalyst with atomically dispersed Ir atoms on the surface of Ni. Overall, the results of AC‐HAADF‐STEM, in situ CO–DRIFTS, and EXAFS characterization collectively support the formation of Ir_1_Ni SAA with atomically dispersed Ir atoms on the surface of Ni nanoparticles, providing experimental evidence that is consistent with the theoretical predictions and DFT calculations.

### 2.6 Mechanism Insight of Hydrogenation of 4‐NS on Ir_1_Ni SAA

**Figure 7:**
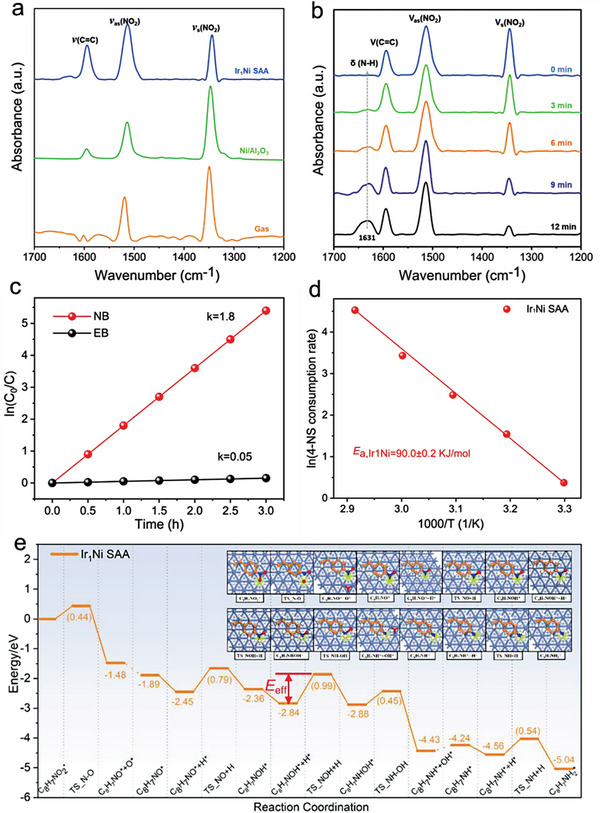
a) In situ FT‐IR spectra of gaseous and chemically adsorbed 4‐NS on Ni and Ir_1_Ni SAA. b) In situ FT‐IR spectra of 4‐NS hydrogenation in the presence of Ir_1_Ni SAA catalyst, recorded within 1700–1200 cm^–1^ by flowing H_2_ as a reaction gas after 0, 3, 6, 9, and 12 min, respectively. c) nitrobenzene and styrene versus reaction time for the hydrogenation reaction of nitrobenzene and styrene mixture (1:1). Reaction conditions: 1 mmol of reactant; 8 mL of solvent (ethanol); 0.03 g of catalyst; 1 Mpa of H_2_, 50 °C. d) Arrhenius plot for Ir_1_Ni SAA catalyst. e) Potential energies profiles and corresponding optimized structures for 4‐NS reduction over Ir_1_Ni(111) surface.

To unravel the microscopic mechanism governing the reduction reaction of 4‐NS, we conducted a comprehensive investigation into the selective hydrogenation process of 4‐NS on the surface of Ir_1_Ni SAA (**Figure** **7**). For the purpose of identifying the interaction between 4‐NS and Ir_1_Ni SAA, we explored it through in situ DRIFTS analysis (Figure 7a,b). In the absence of catalysts, the spectrum of 4‐NS exhibited three peaks at 1601, 1520, and 1350 cm^−1^, attributed to *ν*(C═C), *ν*_as_(NO_2_), and *ν*_s_(NO_2_), respectively.^[^ ^39^, ^40^, ^41^ ^]^ Compared with the gaseous 4‐NS spectrum, *ν*(C═C), *ν*_as_(NO_2_), and *ν*_s_(NO_2_) are red‐shifted by 5, 5, and 2 cm^−1^, respectively. The red shifts indicate the weakening of C═C bonds and N═O bonds, suggesting a strong interaction between both C═C and −NO_2_ groups and surface Ni atoms. In comparison, the peaks of *ν*(C═C) on Ir_1_Ni SAA were located at positions similar to those for Ni nanocrystals, indicating that vinyl tends to adsorb at the Ni sites of Ir_1_Ni SAA. However, in a striking contrast to the pure Ni catalysts, the relative intensity of the *ν*_s_(NO_2_) band at 1343 cm^−1^ is significantly weaker than that of *ν*_as_(NO_2_) at 1513 cm^−1^ on Ir_1_Ni SAA. This is attributed to the cleavage of the N─O bond in −NO_2_ to generate the nitroso intermediate, as indicated by previous studies.^[^ ^42^, ^43^ ^]^ The aforementioned findings validate the synergistic effect of bimetallic sites in Ir_1_Ni SAA, facilitating the activation of the nitro group and enabling its dissociation without the need for H assistance. This alignment is consistent with our DFT‐calculated optimal adsorption configuration and low N─O_1_ bond cleavage barrier. Furthermore, we conducted an in‐depth investigation into the charge distribution of 4‐NS upon adsorption on Ir_1_Ni SAA through DFT calculations (Figure S27 and Table S2, Supporting Information). The results revealed that electrons were transferred from the Ir_1_Ni catalyst surface to 4‐NS, particularly involving the electrons obtained from the nitro group. This further underscores the remarkable activation effect of the Ir─Ni bimetallic site on the nitro group.

To deepen our comprehension of the reaction mechanism, in situ FT‐IR was conducted to monitor the catalytic reaction process of 4‐NS hydrogenation on Ir_1_Ni SAA catalyst sample. Additionally, DFT calculations were performed to understand the reaction process more clearly. As shown Figure 7b, the intensity of *ν*_s_(NO_2_) (1343 cm^−1^) decreases rapidly, accompanied by the appearance of a new peak at 1631 cm^−1^, attributed to the N─H bending vibration. This dynamic change indicates facile reduction of −NO_2_ to −NH_2_. However, a slight increase in *ν*_as_(NO_2_) suggests that N─O_1_ cleavage readily occurs, yet the consumption rate of nitrite intermediates is relatively slow, consistent with our DFT calculation results: the energy barrier for N─O_1_ cleavage is only 0.44 eV, whereas the *C_8_H_7_NOH hydrogenation energy barrier consistently requires 0.99 eV (Figure 7e). Furthermore, no significant decrease is observed in the band of *ν*(C═C) (1595 cm^−1^) after 12 min, indicating that the vinyl groups remain unreduced. This observation also aligns with the DFT calculation results that the vinyl hydrogenation energy barrier is 0.21 eV higher than the N‐O_1_ bond cleavage energy barrier (Figure 7e; Figure S28, Supporting Information). However, the peak intensity of *ν*_s_(NO_2_) and *ν*(C═C) on the surface of the pure Ni catalyst decreased (Figure S29, Supporting Information), indicating the reduction of both nitro and vinyl groups. To further investigate the significant difference in chemoselectivity on Ir_1_Ni SAA, the hydrogenation of a styrene and nitrobenzene mixture (1:1) was also conducted in the presence of Ir_1_Ni SAA catalyst. The results reveal that the change curve of ln (*C*_0_/*C*) with reaction time is a straight line originating from the origin (Figure 7c), indicating that both nitrobenzene and styrene undergo pseudo‐first‐order reactions. Nitrobenzene hydrogenation is kinetically more favorable than styrene hydrogenation (rate constant: 1.80 h^−1^ vs 0.05 h^−1^), emphasizing the preferential selectivity of Ir_1_Ni SAA for nitro reduction. Correspondingly, we employed DFT to calculate the N─O_1_ bond breaking energy barrier of nitrobenzene and the first‐step hydrogenation energy barrier of vinyl of styrene on the Ir_1_Ni SAA surface, yielding values of 0.52 and 0.90 eV (Figure S30, Supporting Information), respectively. This further signifies that Ir_1_Ni SAA exhibits high activity and remarkable chemical selectivity for nitro reduction. Through the calculation of the selective hydrogenation reaction path of 4‐NS, the results reveal that the reaction energy trend of 4‐NS on Ir_1_Ni SAA is consistent with that on Ru_1_M_host_ SAAs. In this pathway, the hydrogenation of C_8_H_7_NOH is identified as the rate‐controlling step of the entire reaction, with a hydrogenation energy barrier of 0.99 eV (Figure 7e). To further unveil the activity of the Ir_1_Ni SAA catalyst, we measured its apparent activation energy, yielding a value of 90 ± 0.2 kJ mol^−1^ (Figure 7d). Notably, the experimentally measured apparent activation energy is very close to the DFT‐calculated effective energy barrier (*E*_eff_ = 0.99 eV ≈95.52 kJ mol^−1^). This strongly indicates that the hydrogenation of C_8_H_7_NOH is a key step controlling the selective hydrogenation of 4‐NS, consolidating the reliability of our knowledge of reaction pathways and mechanisms. The distinctive interface structure between Ir single atoms and the Ni matrix, combined with the synergistic effect of the host–guest metals in Ir_1_Ni SAA, results in outstanding activity and chemical selectivity for the hydrogenation of 4‐NS.

## 3 Conclusion

In summary, 15 SAAs were studied and evaluated as catalysts for the chemoselective hydrogenation of 4‐NS to produce 4‐AS using DFT calculations. The geometric effect analysis revealed that a larger difference in atomic radii between the host and guest metals leads to shorter M_1_─M_host_ bonds in the SAAs, which facilitates the activation of nitro groups. The investigation of the reaction mechanism identified two key steps: N−O_1_ bond activation and C_8_H_7_NOH hydrogenation. For the N−O_1_ bond scission, the doping of Ru and Ir forms short M_1_−M_host_ bonds, which is beneficial to the activation of the N−O_1_ bond, the N−O_1_ bond breaking energy barrier is only about 0.40 eV. However, for C_8_H_7_NOH hydrogenation, the hydrogenation energy barrier mainly influenced by the host metal, showed minimal impact from the addition of different noble metals. Based on the activation of N−O_1_ bond, C_8_H_7_NOH hydrogenation and vinyl hydrogenation, two promising SAAs (Ru_1_Ni and Ir_1_Ni) for the selective hydrogenation of 4‐NS were identified. Furthermore, experimental evaluation of the synthesized catalysts confirmed the excellent catalytic performance of Ir_1_Ni, exhibiting high conversion (>98%) and selectivity (>98% for 4‐AS). Characterization techniques including XRD, AC‐HAADF‐STEM, XPS, and EXAFS provided further evidence of the atomically dispersed Ir atoms on the surface of Ni nanoparticles in Ir_1_Ni SAA. The reciprocal validation between in situ FT‐IR experiments and DFT calculations provides further evidence that the distinctive host–guest metal interaction at the Ir–Ni interface site of Ir_1_Ni SAA empowers it with exceptional performance in the selective hydrogenation of 4‐NS. This work highlights the potential of rational design and precise synthesis of SAA catalysts with controlled selectivity.

## 4 Experimental Section

### DFT Computational

The first‐principle calculation was performed by using the Vienna ab initio simulation package (VASP 5.4.4).^[^ ^44^, ^45^ ^]^ The core electrons were modeled using the projector augmented wave (PAW) approach.^[^ ^46^, ^47^ ^]^ The exchange‐correlation potential was approximated with the PBE‐D3^[^ ^48^ ^]^ functional. In order to verify the accuracy of the DFT‐D3 functional calculation results, PBE^[^ ^49^ ^]^ and PBEsol^[^ ^50^ ^]^ were selected as the comparison functionals. The functionals were applied to for calculate both the structural (lattice constant), energetic (binding energy) properties (Table S3 and Figure S31, Supporting Information). All these three functional gave the same trend when predicting the binding energy. While PBE‐D3 demonstrating superior accuracy in determining lattice constants. Therefore, the PBE‐D3 functional was chosen for the subsequent calculations. The transition states were first approached using the climbing image nudged elastic band (CI‐NEB),^[^ ^51^ ^]^ or the DIMER method^[^ ^52^ ^]^ More detailed computational methods are described in the Supporting Information.

### Catalyst Preparation

As precursors, hierarchical NiAl‐LDHs was synthesized by in situ growth method.^[^ ^35^, ^53^ ^]^ Specifically, Ni/Al_2_O_3_ was prepared by reducing NiAl‐LDHs (0.3 g) in a H_2_/N_2_ (10/90, v/v; 35 mL min^−1^) stream at 500 °C for 4 h (heating rate: 2 °C min^−1^) to prepare. A set of M_1_Ni/Al_2_O_3_ (M_1_ = Pt, Pd, Rh, Ir, and Ru) SAA were prepared through a galvanic replacement method. Taking Ir_1_Ni SAA as an example, the fresh Ni/Al_2_O_3_ sample (0.2 g) was dispersed in 30 mL purified water, then H_2_IrCl_6_ solution (0.07 mmol L^−1^) was slowly added, and vigorously stirred for 60 min under the protection of a N_2_ atmosphere. The 0.6% Ir_1_Ni sample was obtained by centrifuging the precipitate obtained above, washing with purified water, and drying in a cacuum oven at 50 °C for 24 h. The Ru_1_Ni, Rh_1_Ni, Pd_1_Ni and Pt_1_Ni samples were prepared via the same method with tuning the desired amount of corresponding metal precursor solution. Details are described in the Supporting Information.

### Catalytic Test

During an experiment, the 4‐NS was added into a stainless‐steel reactor (25 mL) at 1 mPa of H_2_, 50 °C, 4 h. First, carefully add the 4‐NS (substrate 1 mmol), ethanol (solvent 8 mL) and catalyst (0.03 g) to a 25 mL stainless‐steel autoclave. Subsequently, the reactor was completely purged for five times with 2.0 MPa hydrogen (>99.999%), followed by pressurized and sealed with H_2_ to 1.0 MPa. The reaction was performed at 50 °C with a constant stirring speed of 700 rpm. The resulting products were identified by GC‐MS, and quantitatively analyzed using a Shimadzu GC−2014C gas chromatograph system outfitted a GSBP−INOWAX capillary column (30m × 0.25mm × 0.25 mm) and an FID detector. The conversion of 4‐nitrostyrene and the selectivity of products were determined as follows:

(1) $$\begin{array}{c} \mathrm{Conversion}\left(\%\right)=\left(1-\frac{\mathrm{Mole}\;\mathrm{number}\;\mathrm{of}\;4-\mathrm{NS}\;\mathrm{after}\;\mathrm{reaction}}{\mathrm{Initial}\;\mathrm{mole}\;\mathrm{number}\;\mathrm{of}\;4-\mathrm{NS}\;\mathrm{fed}}\right)\times 100\% \end{array}$$

(2) $$\mathrm{Selectivity}\left(\%\right)\;=\;\frac{\mathrm{Mole}\;\mathrm{number}\;\mathrm{of}\;\mathrm{one}\;\mathrm{product}}{\mathrm{Total}\;\mathrm{mole}\;\mathrm{number}\;\mathrm{of}\;4-\mathrm{NS}\;\mathrm{converted}}\;\times \;100\%\;$$

## Conflict of Interest

The authors declare no conflict of interest.

## Supporting information

Supporting Information

## Data Availability

The data that support the findings of this study are available from the corresponding author upon reasonable request.
