# Supplementary material for: Rational Design and Precise Synthesis of Single‐Atom Alloy Catalysts for the Selective Hydrogenation of Nitroarenes
Source: Adv Sci (Weinh). 2024 Apr 10;11(23):2304908. doi: 10.1002/advs.202304908 (PMC11187892; doi:10.1002/advs.202304908)
Supplement: Supplementary file 1 — Supporting Information [file ADVS-11-2304908-s001.pdf]

ADVANCED SCIENCE
Open Access

Supporting Information

for *Adv. Sci.*, DOI 10.1002/advs.202304908

Rational Design and Precise Synthesis of Single-Atom Alloy Catalysts for the Selective Hydrogenation of Nitroarenes

Haisong Feng, Wei Liu, Lei Wang, Enze Xu, Donghui Pang, Zhen Ren, Si Wang, Shiquan Zhao, Yuan Deng, Tianyong Liu, Yusen Yang, Xin Zhang*, Feng Li and Min Wei*

Supporting Information

Rational design and precise synthesis of single-atom alloy catalysts for the selective hydrogenation of nitroarenes

Haisong Feng,[1] Wei Liu,[1] Lei Wang,[1] Enze Xu,[1] Donghui Pang,[1] Zhen Ren,[1] Si Wang,[1] Shiquan Zhao,[1] Yuan Deng,[1] Tianyong Liu,[1] Yusen Yang*,[1,2] Xin Zhang*,[1,2] Feng, Li,[1] and Min Wei[1,2]

[1] State Key Laboratory of Chemical Resource Engineering, Beijing Advanced Innovation Center for Soft Matter Science and Engineering, Beijing University of Chemical Technology, Beijing 100029, P. R. China

[2] Quzhou Institute for Innovation in Resource Chemical Engineering, Quzhou 324000, P. R. China

Author Information

* Corresponding authors. Tel.: +8610-64412131; Fax: +8610-64425385.

E-mail addresses: yangyusen@mail.buct.edu.cn (Y. Yang); zhangxin@mail.buct.edu.cn (X. Zhang).

Supplementary methods

DFT computational methods. The first-principle calculation was performed by using the Vienna *ab initio* simulation package (VASP 5.4.4)[1,2]. The projector augmented wave (PAW) method was used to describe the core electrons[3,4]. PBE-D3[5,6], PBE[5] and PBEsol[7] functionals were used to calculate both the structural properties (lattice constant) and the energetic properties (binding energy (E_b)). Firstly,

the lattice constants of Cobalt (Co), Nickel (Ni), and Copper (Cu) and the results are summarized as Table S3. Evidently, the findings underscore that the lattice constants derived from PBE-D3 align more closely with experimental values. Hence, we assert that among these three functionals, PBE-D3 emerges as the optimal choice for the meticulous computation of M_1Co, M_1Ni and M_1Cu SAAs. Secondly, we conducted an assessment of the binding energies of 4-nitrostyrene on $Ru_1Co(111)$, $Ru_1Ni(111)$, and $Ru_1Cu(111)$ surfaces. The results show that compared with the calculation results of PBE-D3 functional, the PBE functional consistently displays a propensity to underestimate binding energies. Conversely, the PBEsol functional tends to magnify the binding energy of 4-nitrostyrene on $Ru_1Co(111)$ and $Ru_1Ni(111)$ surfaces, while concurrently exhibiting an inclination to underestimate the binding energy on $Ru_1Cu(111)$. Even though the absolute value changes, the trend are exactly same that the 4-nitrostyrene adsorbs strongest on $Ru_1Co(111)$ and weakest on $Ru_1Cu(111)$, as shown in Figure S31. All three of these functionals showed consistent trends in predicting binding energies. While PBE-D3 demonstrating superior accuracy in determining lattice constants. Therefore, the PBE-D3 functional was chosen for the subsequent calculations. A 3×3×1 Monkhorst-Pack *k* point mesh and 400 eV cutoff energy for the plane-wave basis were employed for the geometry optimizations. The convergence criterion for the total energy self-consistent iterations was set to 10^{-4} eV, and the geometry optimization stops when the total force was less than 0.05 eV/Å. The energy barriers of transient states were determined using the climbing image nudged elastic band (CI-NEB) method[8], or the DIMER method,[9] and vibrational frequency analysis was conducted to confirm only one imaginary frequency in each transition state.

Co(111), Ni(111) and Cu(111) surfaces were represented by *p*(5×5) periodic unit cells with three atomic layers, and a vacuum space of 15 Å was added in the *z* direction to minimize the interaction between the periodically repeated slabs or adsorbates in the direction normal to the slab. The SAA surfaces were built by substituting one host atom (Co, Ni or Cu) on the topmost layer with one noble

metal atom. During all the optimizations, the bottommost layer of SAA was fixed, while the top two layers were fully relaxed to participate in reactions.

The binding energy (E_b) of 4-NS on SAAs surfaces and the activation energy barrier (E_a) of 4-NS reduction are calculated by

$$E_b = E_A + E_M - E_{A/M} \quad \text{(S1)}$$

and

$$E_a = E_{TS} - E_{IS} \quad \text{(S2)}$$

where $E_{A/M}$, E_A, E_M, E_{TS} and E_{IS} represent the energies of adsorbed system, substrate, adsorbate species, transition state (TS) and initial state (IS), respectively.

The *d* band center[10] of SAAs is calculated by

$$\varepsilon_d = \frac{\int_{-\infty}^{+\infty} n_d(\varepsilon)\varepsilon d_\varepsilon}{\int_{-\infty}^{+\infty} n_d(\varepsilon) d_\varepsilon} \quad \text{(S3)}$$

where ε is the energy with respect to the Fermi level, and $n_d(\varepsilon)$ is the electronic density of states.

The Bader Charge analysis was performed *via* Henkelman programme based on near-grid algorithm with refine-edge method[11–13] The charge density differential analysis was performed by using visualization software VESTA[14].

Chemicals and materials. Analytical reagents used in the experiments were bought from Sigma Aldrich: $Al_2(SO_4)_3\cdot18H_2O$, sodium tartrate, $CO(NH_2)_2$, NH_4NO_3, $Ni(NO_3)_2\cdot6H_2O$, $RuCl_3$, $RhCl_3$, $(NH_4)_2PdCl_4$, H_2IrCl_6, H_2PtCl_6, ethanol, 4-nitrostyrene (4-NS), 4-aminostyrene, 4-nitroethylbenzene, 4-aminoethylbenzene. Purified water was adopted in all the experiments.

Synthesis of catalysts. As a precursor, hierarchical NiAl-LDHs was synthesized by *in situ* growth method reported previously by our group[15]. Afterwards, NiAl-LDHs (0.3 g) was reduced in a H_2/N_2 (10/90, v/v; 35 mL·min^{-1}) stream at 500 °C for 4 h (heating rate: 2 °C·min^{-1}) to prepare amorphous

Al_2O_3 supported monometallic Ni sample (Ni/Al_2O_3). The supported M_1Ni bimetallic samples were synthesized by a galvanic replacement method. Taking Ir_1Ni SAA as an example, the fresh Ni/Al_2O_3 sample (0.2 g) was dispersed in 30 mL purified water, followed by slowly adding H_2IrCl_6 solution (0.07 mmol·L^{-1}) and stirring vigorously for 60 min under the protection of a N_2 atmosphere. The obtained precipitation was centrifugated, washed with purified water, and dried for 24 h in vacuum oven at 50 °C to obtain 0.6% Ir_1Ni sample. The Ru_1Ni, Rh_1Ni, Pd_1Ni and Pt_1Ni samples were prepared via the same method with tuning the desired amount of corresponding metal precursor solution. Before the catalytic evaluation, the as-synthesized samples were pre-reduced in a H_2/N_2 flow (10/90, v/v) at 300 °C (heating rate: 2 °C·min^{-1}) for 1 h, followed by cooling to the room temperature in N_2. As references, Ir/Al_2O_3 samples were prepared by a deposition precipitation method and reduced at 300 °C (H_2/N_2 flow: 10/90, v/v; heating rate: 2 °C·min^{-1}) for 3 h.

Characterizations. The X-ray diffraction (XRD) experiments were carried out on Bruker DAVINCI D8 ADVANCE diffractometer with a Cu Kα radiation source (40 kV and 40 mA). Scanning electron microscope (SEM) images were displayed by using a Zeiss Supra 55 electron microscope. Transmission electron microscopy (TEM) characterizations were performed on a JEOL JEM-2010 high-resolution transmission electron microscope. FEI Titan Cube Themis G2 300 and JEOL JEMARM200F instruments with a spherical aberration corrector and energy-dispersive X-ray spectroscopy (EDS) system were adopted to perform aberration-corrected high angle annular dark-field scanning transmission electron microscopy (AC−HAADF−STEM) and EDS mapping measurements. X-ray absorption fine structure spectroscopy (XAFS) at Ir K-edge and Ni K-edge were measured at the beamline 1W1B and 1W2B of the Beijing Synchrotron Radiation Facility (BSRF), Institute of High Energy Physics (IHEP), Chinese Academy of Sciences (CAS). A Ir foil reference was

scanned simultaneously for energy calibration. The raw data were processed using the Athena interface of the Demeter software package.[16] Wavelet transformation for the Ir L3-edge XAFSsignals was employed based on Morlet wavelets.[17] For the CO-DRIFTS experiment, about 50 mg of sample was carefully put into the support sink of diffuse reflectance cell firstly. Subsequently, the sample was pre-reduced in a H_2/He flow (1/19, v/v; 30 mL·min^{-1}) at 300 °C (heating rate: 5 °C·min^{-1}) for 1 h, followed by cooling to the room temperature in a high purity He stream, and collecting background signal. Afterwards, the CO/He (1/19, v/v; 30 mL·min^{-1}) was purged into the cell, and then DRIFTS spectra were collected until the adsorption spectrums kept unchanged. Finally, the gas flow was switched to a pure He stream to collect CO chemisorption spectra. *In situ* FT-IR measurements of 4-NS adsorption and surface reaction were performed using a transmission reactor. The sample (20 mg) was pressed into self-supporting wafer with a diameter of 13 mm, followed by a pretreatment under the same conditions. After the sample was cooled down to 50 °C in He stream, 4-NS was introduced into the reactor for 30 min; and He was purged to remove the physically adsorbed molecule followed by collection of IR signals. Subsequently, the spectra for hydrogenation process were collected per 60 s after the introduction of H_2 (flow rate: 30 mL·min^{-1}).

Catalytic test. Firstly, substrate (4-NS, 1 mmol), solvent (ethanol, 8 ml) and catalyst (0.03 g) were carefully added to a 25 mL stainless-steel autoclave. Subsequently, the reactor was purged completely with 2.0 MPa hydrogen (>99.999%) for 5 times, followed by pressurized and sealed with H_2 to 1.0 MPa. The reaction was carried out at 50 °C with a constant stirring speed of 700 rpm. After the reaction is over, the resulting products were identified by GC-MS, and quantitatively analyzed using a Shimadzu GC−2014C gas chromatograph system outfitted a GSBP−INOWAX capillary column (30m×0.25mm×0.25mm) and an FID detector. The conversion of 4-nitrostyrene and the selectivity of

products were determined as follows:

$$\text{Conversion}(\%) = \left(1 - \frac{\text{Mole number of 4-NS after reaction}}{\text{Initial mole number of 4-NS fed}}\right) \times 100\% \qquad (4)$$

$$\text{Selectivity}(\%) = \frac{\text{Mole number of one product}}{\text{Total mole number of 4-NS converted}} \times 100\% \qquad (5)$$

Supplementary figures

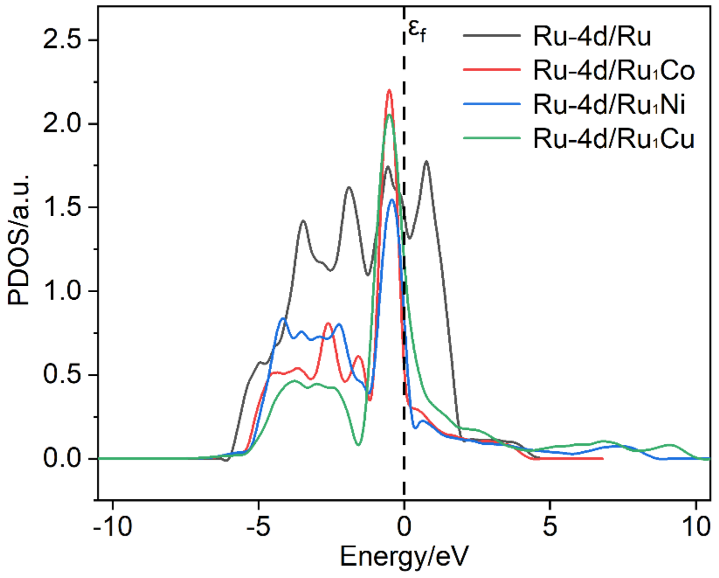

Figure S1 Projected density of states (PDOS) for Ru_1M_{host} SAA.

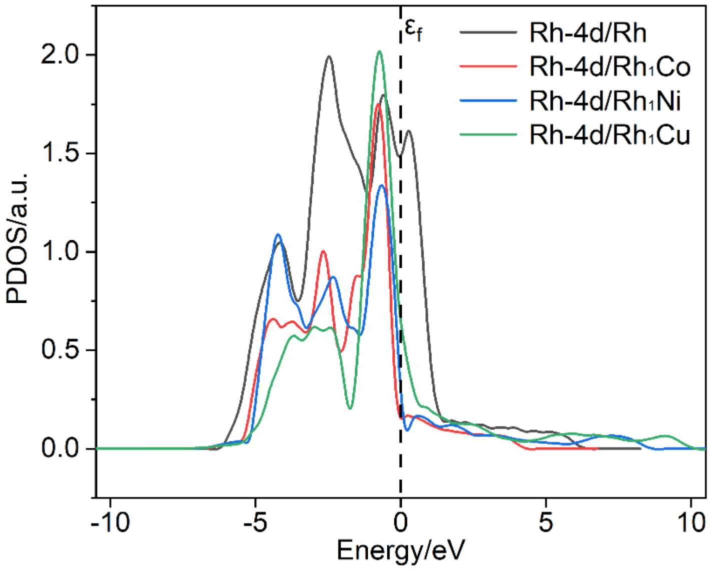

Figure S2 Projected density of states (PDOS) for Rh_1M_{host} SAA.

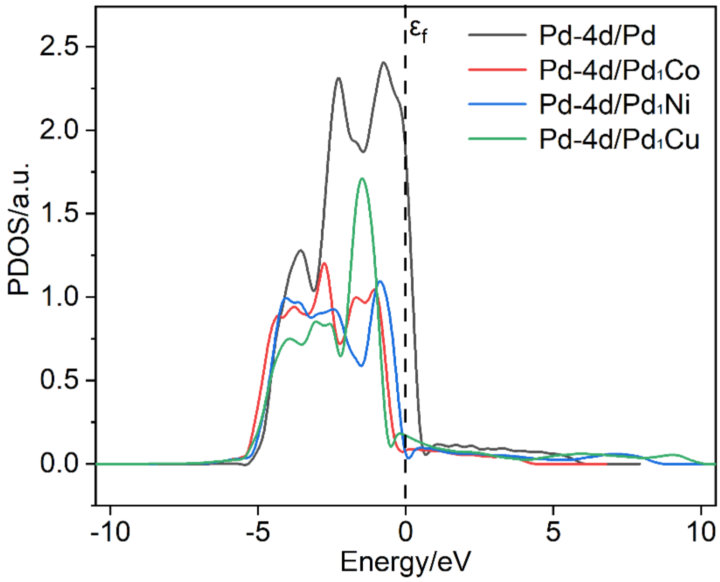

Figure S3 Projected density of states (PDOS) for Pd_1M_{host} SAA.

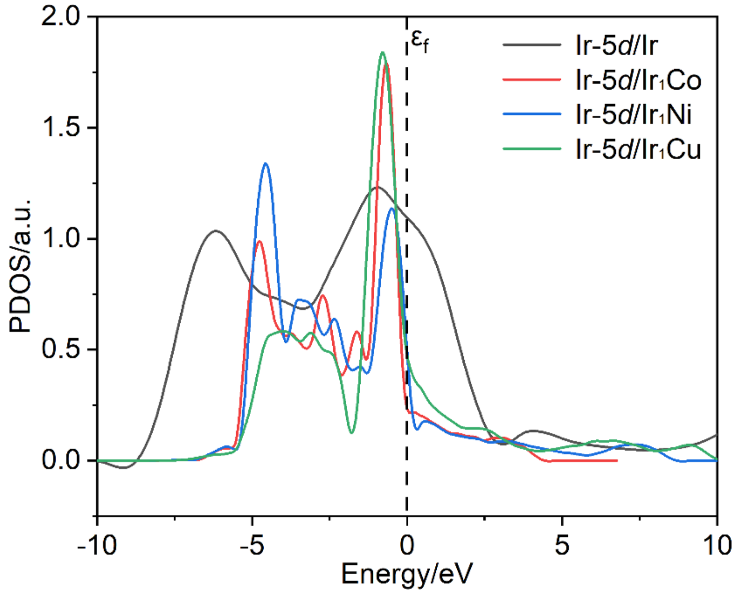

Figure S4 Projected density of states (PDOS) for Ir_1M_{host} SAA.

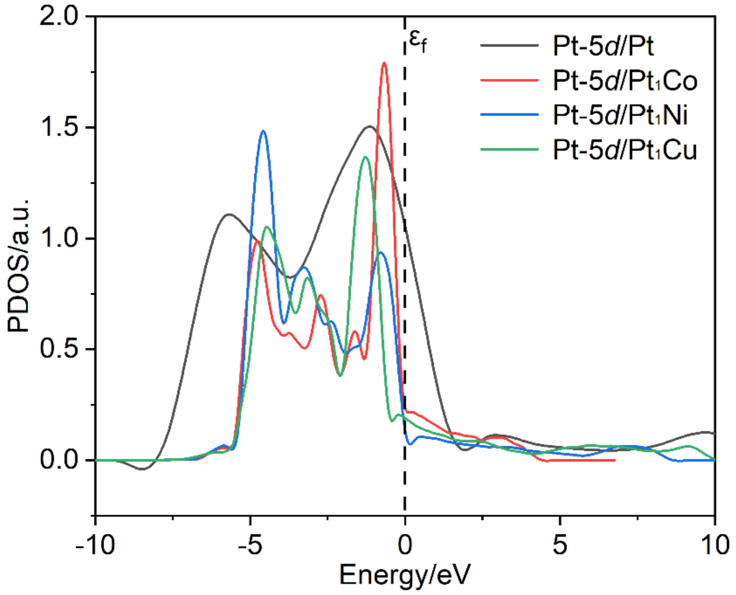

Figure S5 Projected density of states (PDOS) for Pt_1M_{host} SAA.

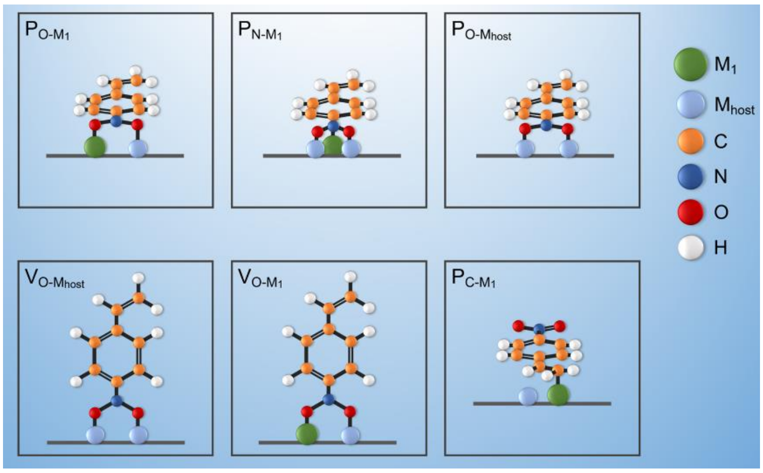

Figure S6 Schematic diagram of the adsorption configuration of 4-nitrostyrene on the surfaces of SAA.

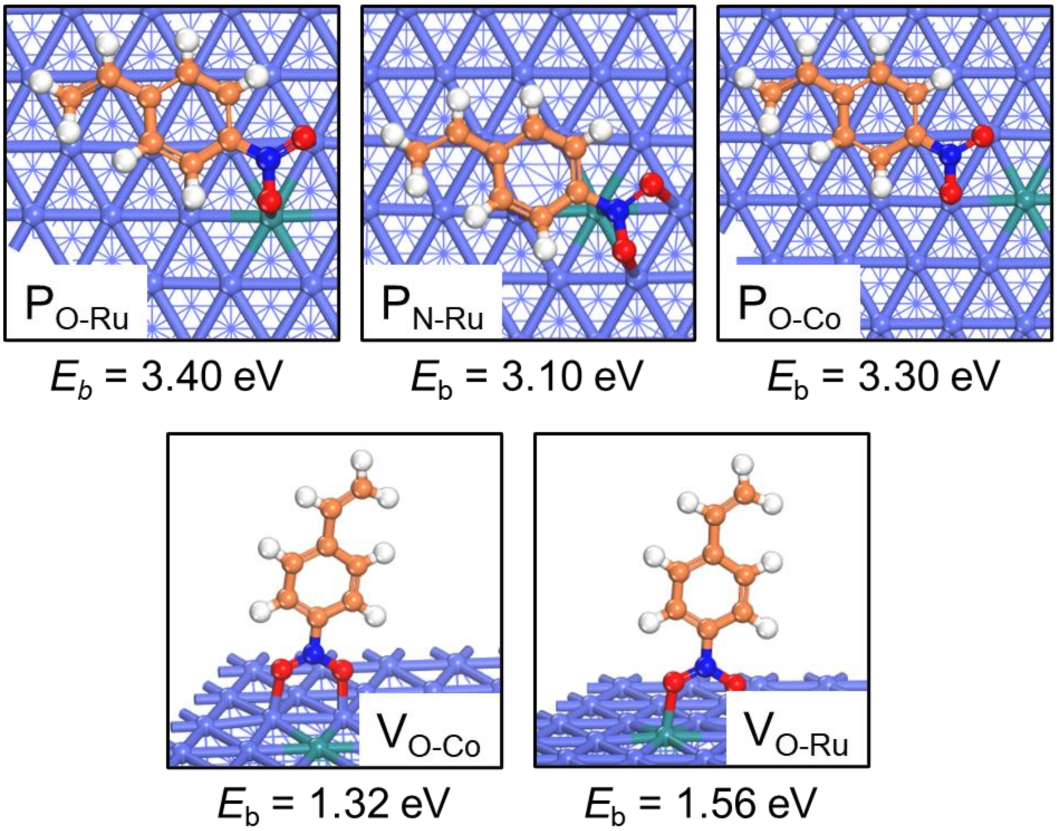

Figure S7 Adsorption configurations and corresponding binding energies of 4-NS on Ru_1Co SAA.

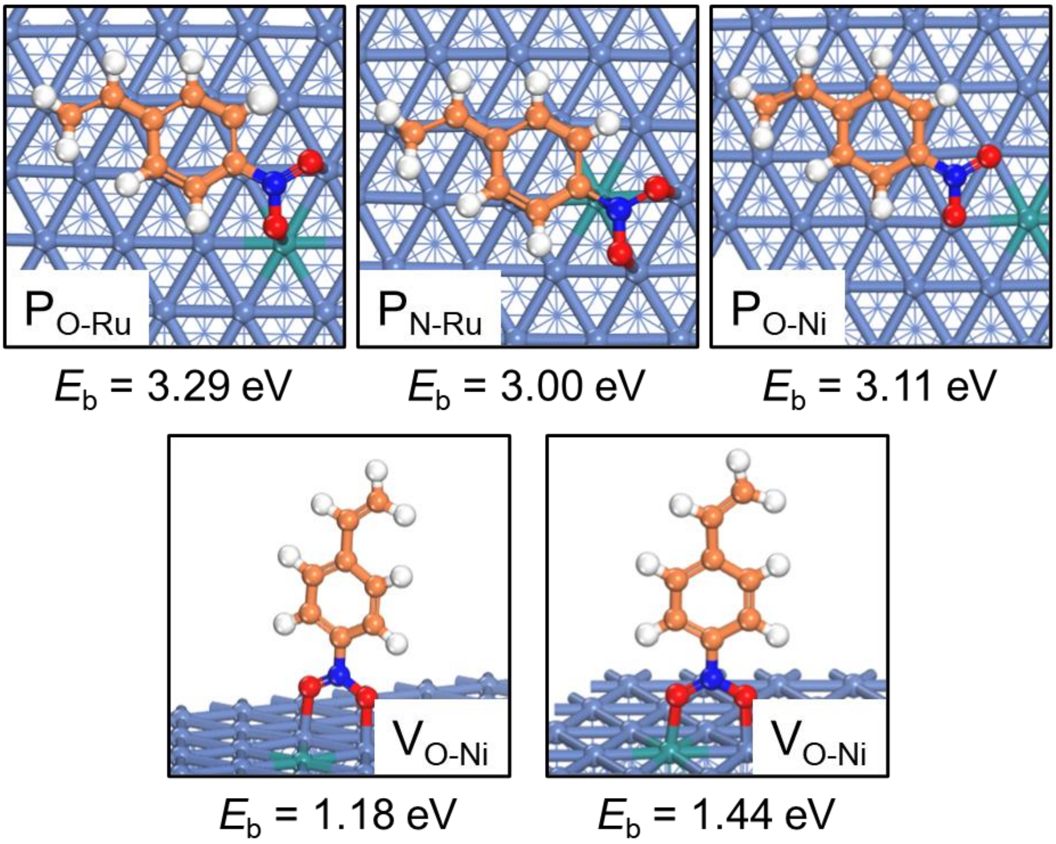

Figure S8 Adsorption configurations and corresponding binding energies of 4-NS on Ru_1Ni SAA.

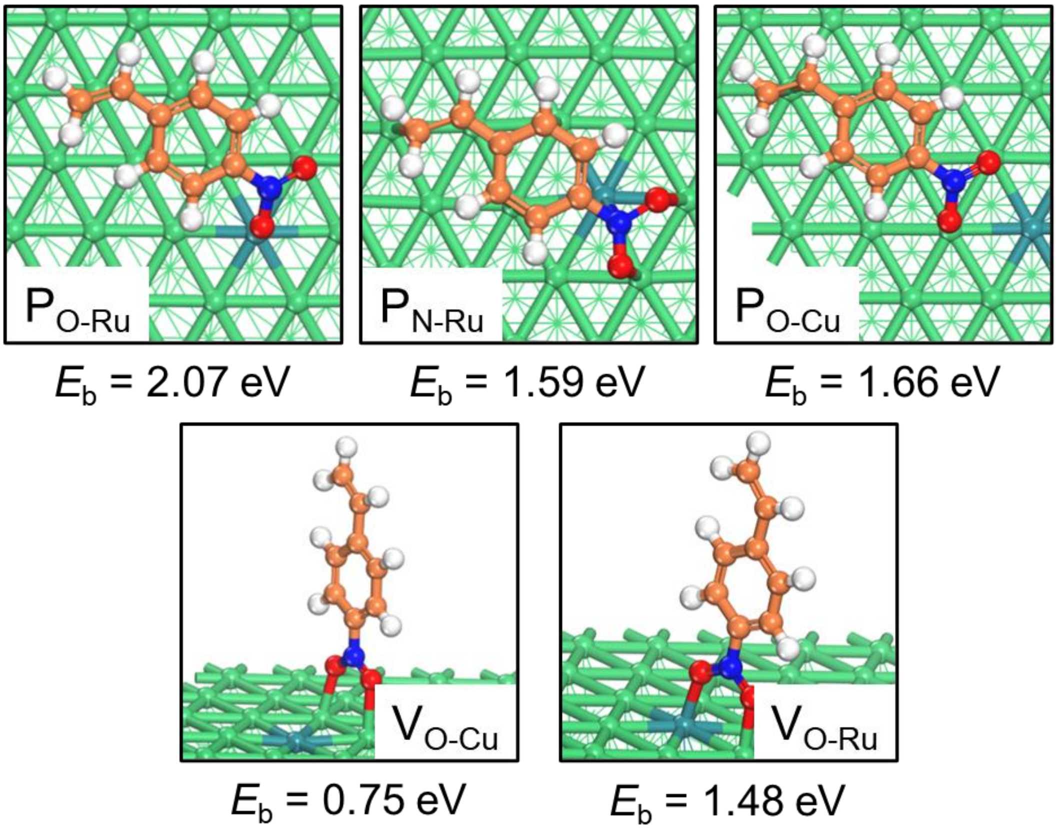

Figure S9 Adsorption configurations and corresponding binding energies of 4-NS on Ru_1Cu SAA.

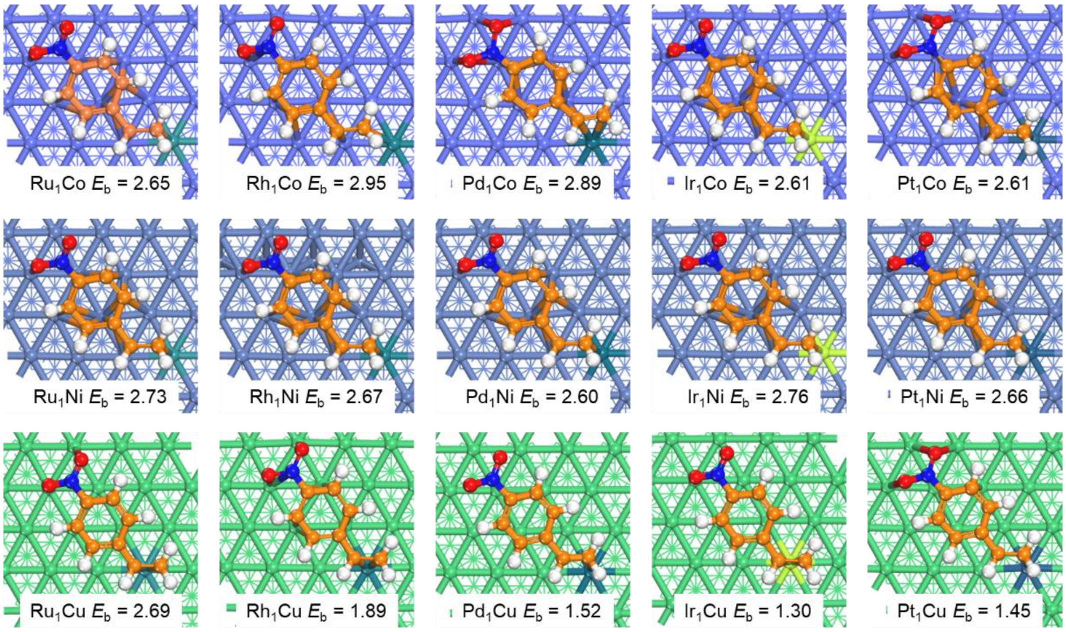

Figure S10 The adsorption configuration and corresponding binding energy of vinyl adsorbed on single atom sites.

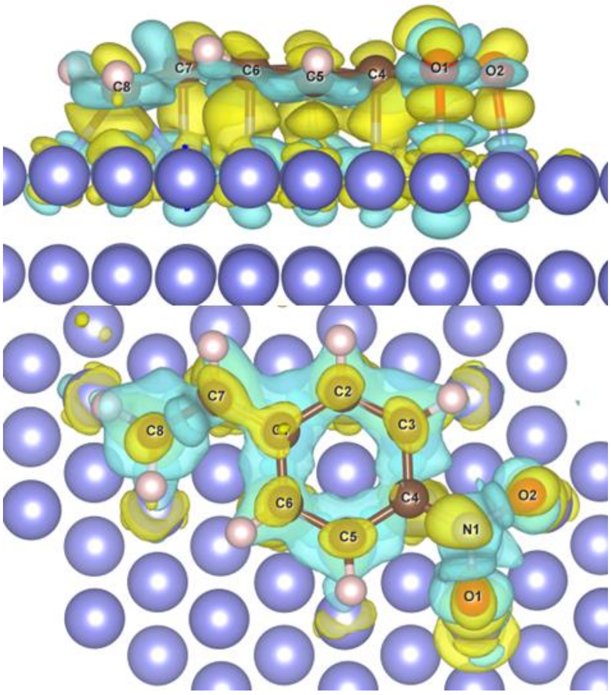

Figure S11 Charge difference distribution for 4-NS adsorption over $Ru_1Co(111)$ surface (the cutoff of the density-difference isosurface is 0.003 e Å$^{-3)}$.

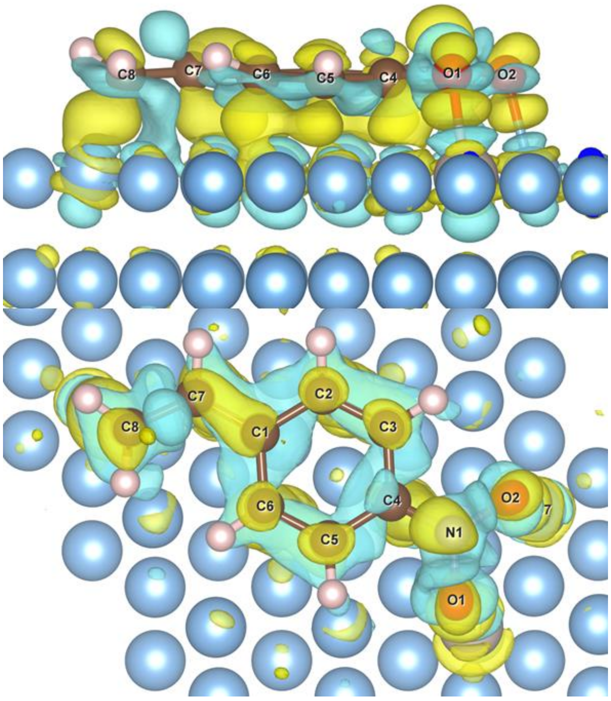

Figure S12 Charge difference distribution for 4-NS adsorption over $Ru_1Ni(111)$ surface (the cutoff of the density-difference isosurface is 0.003 e $Å^{-3}$).

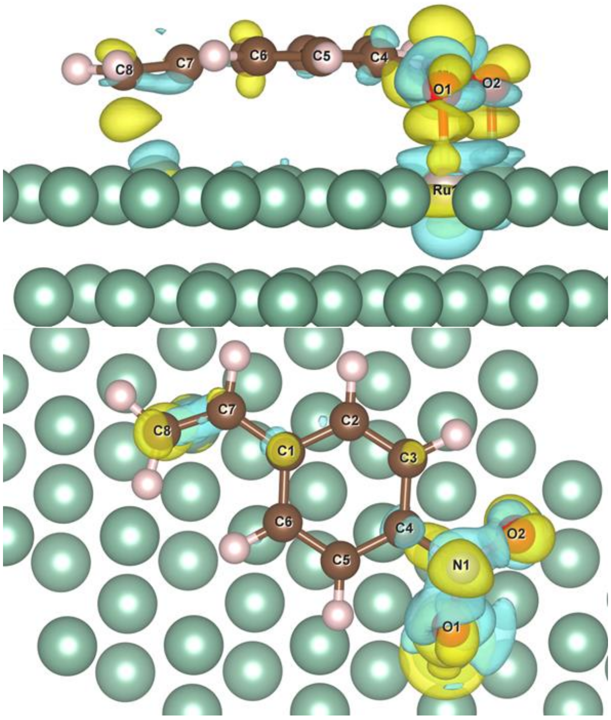

Figure S13 Charge difference distribution for 4-NS adsorption over $Ru_1Cu(111)$ surface (the cutoff of the density-difference isosurface is 0.003 e $Å^{-3}$).

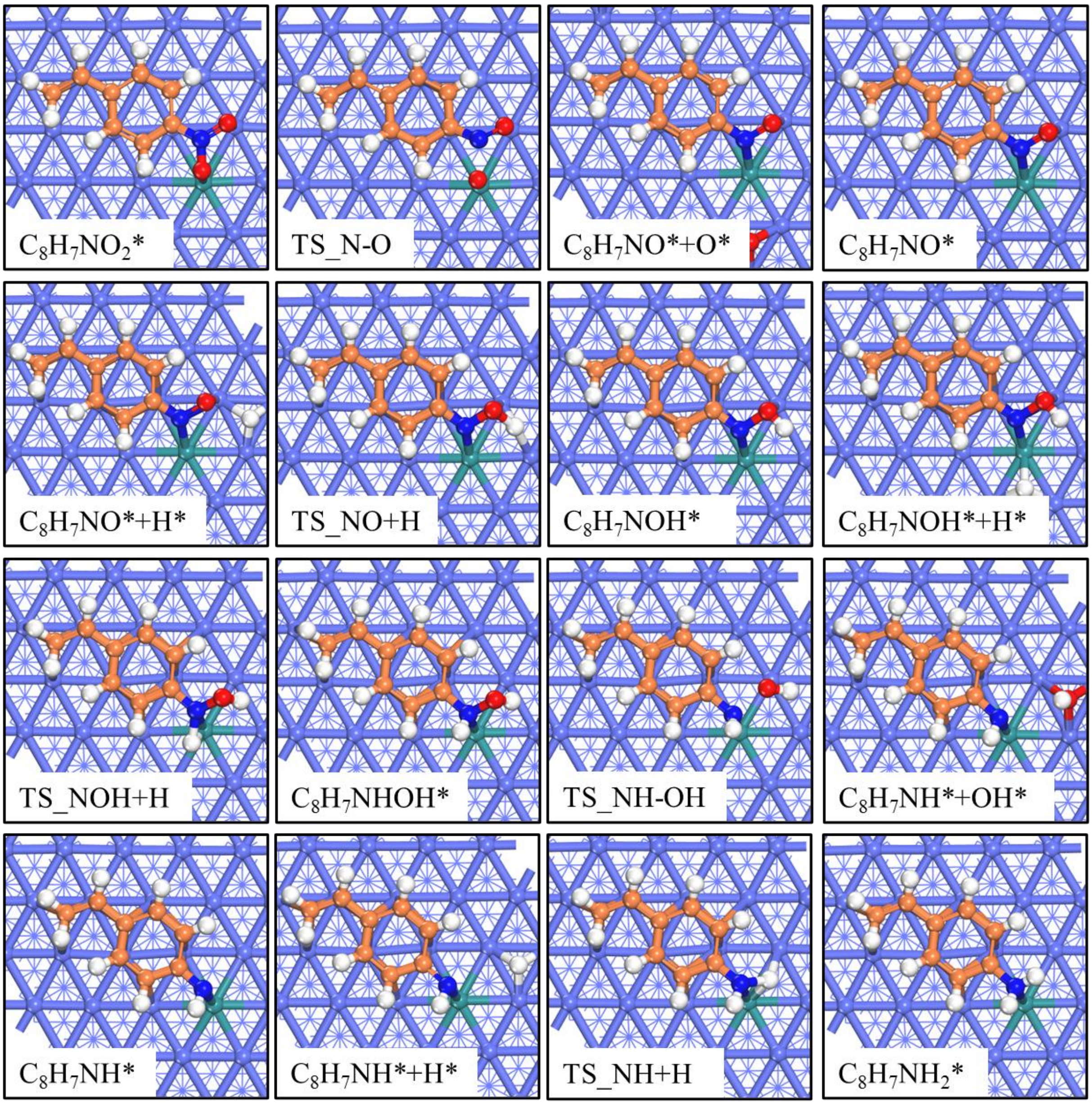

Figure S14 The structures of intermediates and transition state for the 4-NS reduction on $Ru_1Co(111)$.

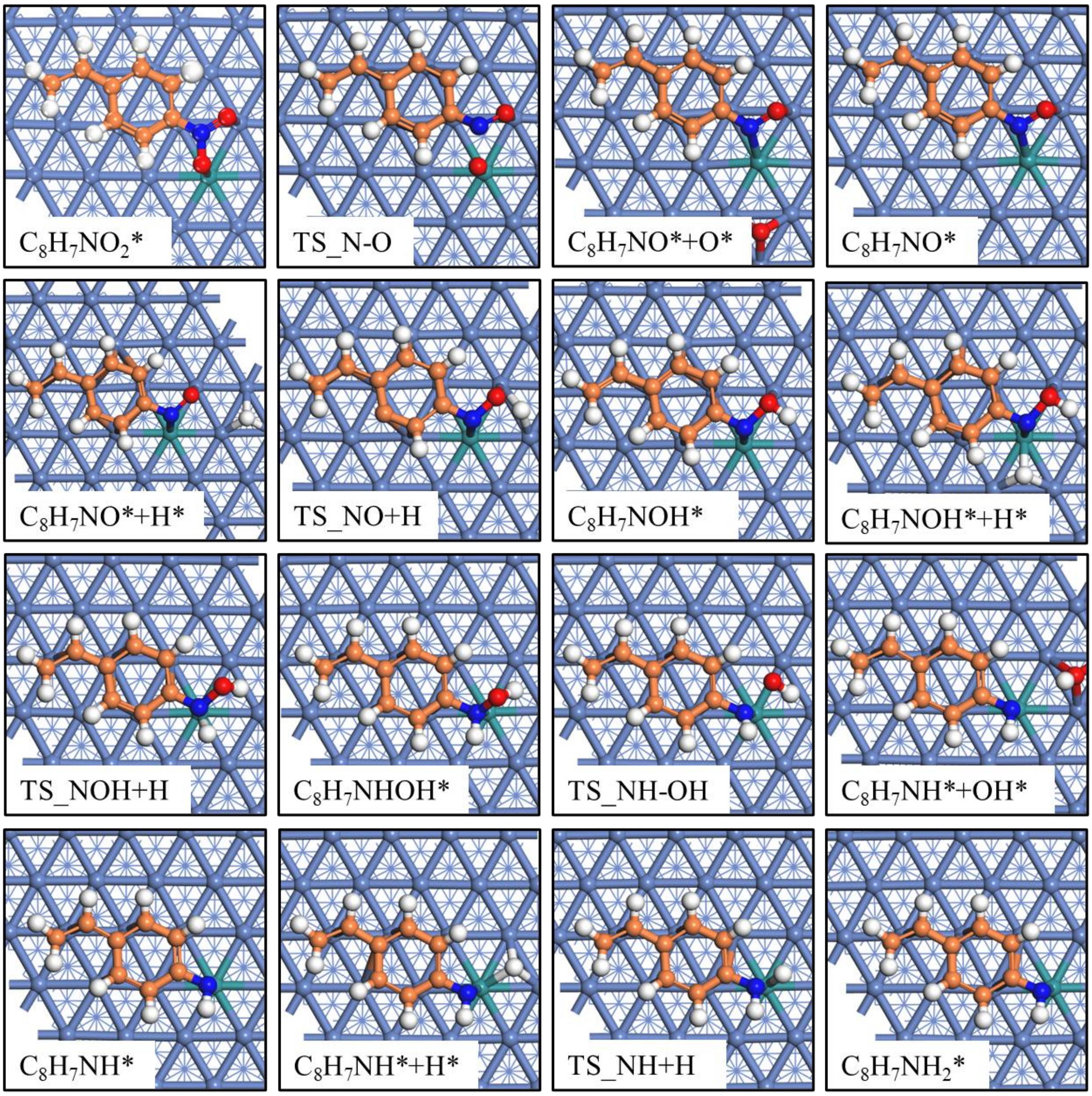

Figure S15 The structures of intermediates and transition state for the 4-NS reduction on $Ru_1Ni(111)$.

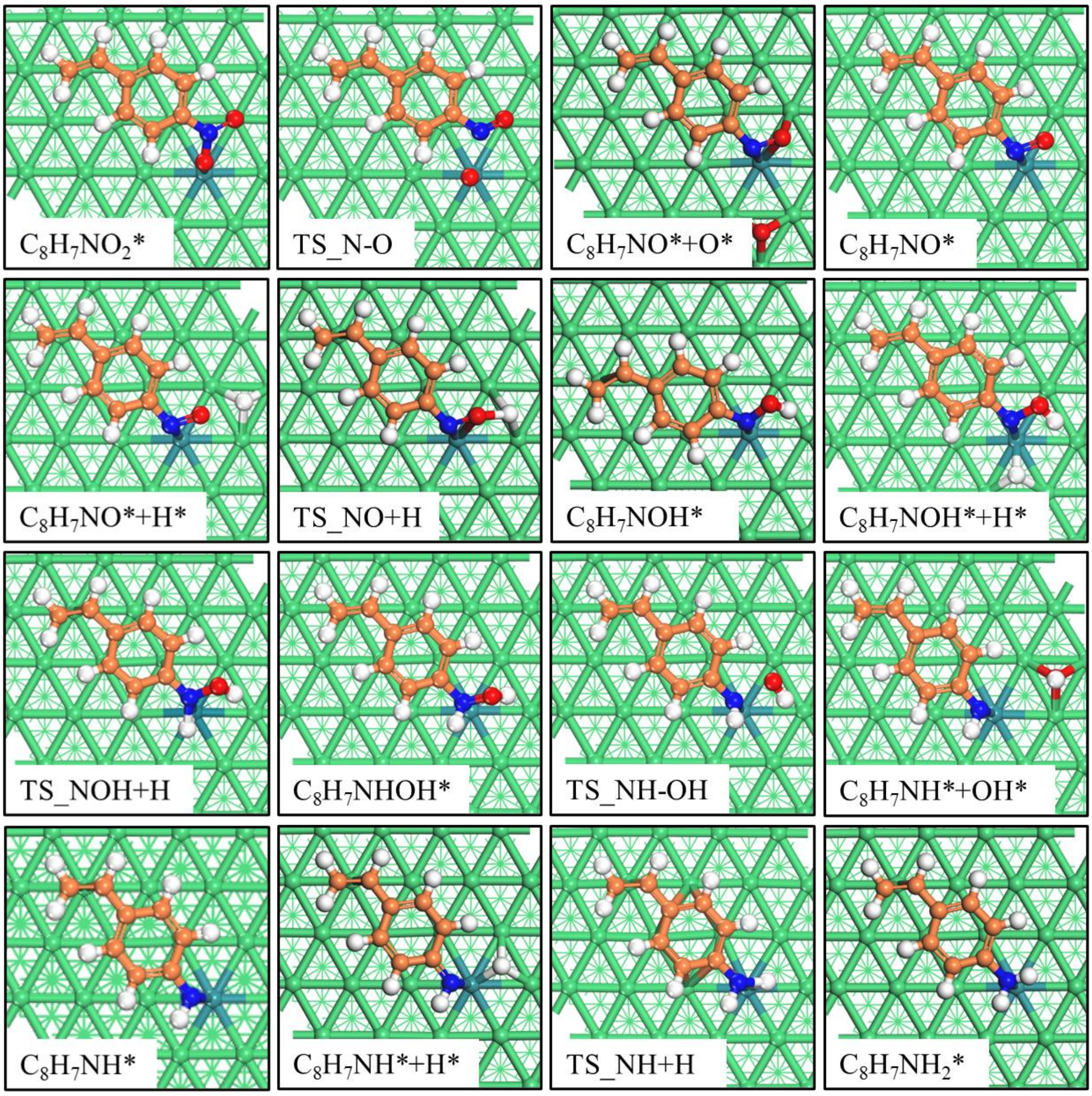

Figure S16 The structures of intermediates and transition state for the 4-NS reduction on $Ru_1Cu(111)$.

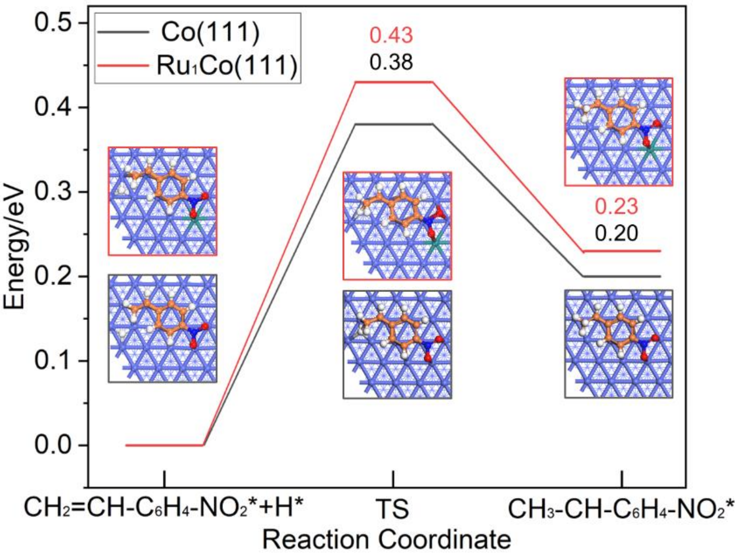

Figure S17 Potential energies profiles and corresponding optimized structures for C=C hydrogenation over Co(111) and $Ru_1Co(111)$ surface.

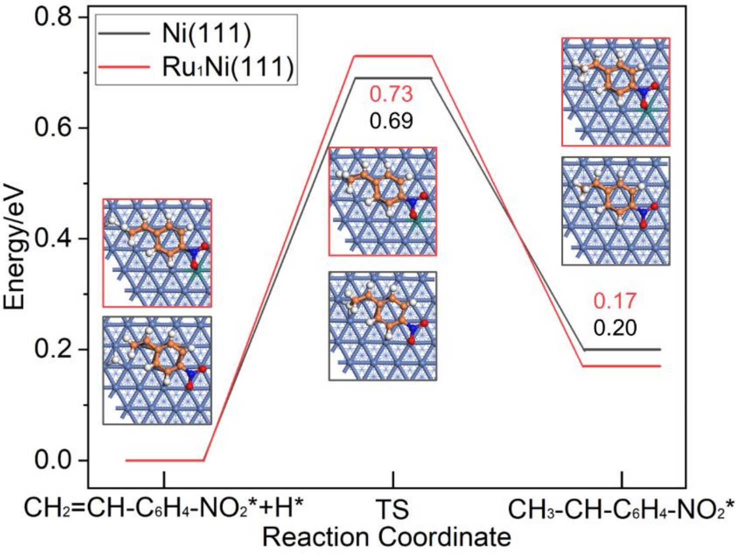

Figure S18 Potential energies profiles and corresponding optimized structures for C=C hydrogenation over Ni(111) and $Ru_1Ni(111)$ surface.

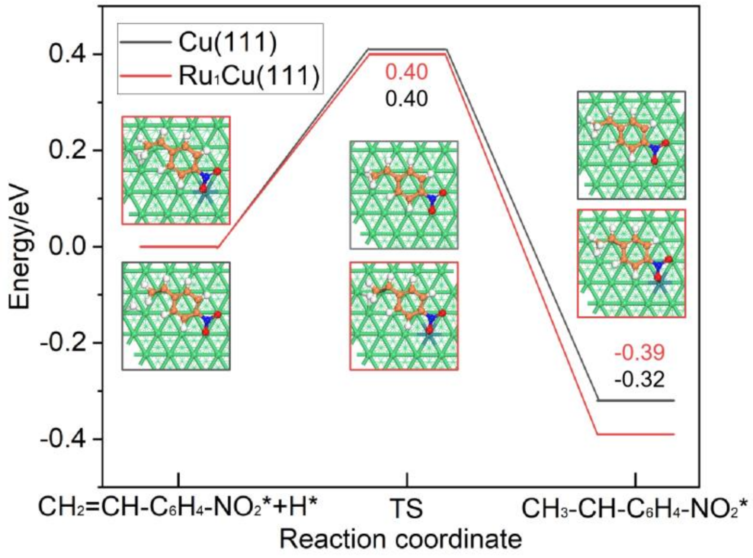

Figure S19 Potential energies profiles and corresponding optimized structures for C=C hydrogenation over Cu(111) and $Ru_1Cu(111)$ surface.

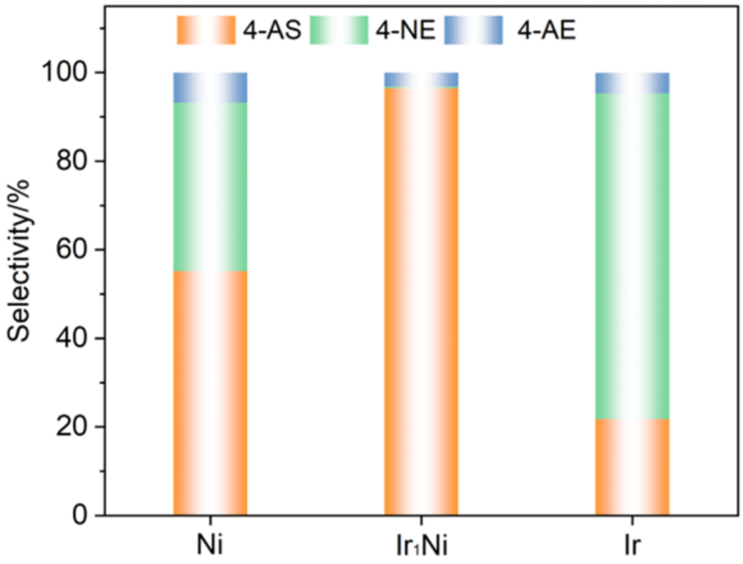

Figure S20 Product distribution in the presence of monometallic Ni, Ir and Ir_1Ni catalysts.

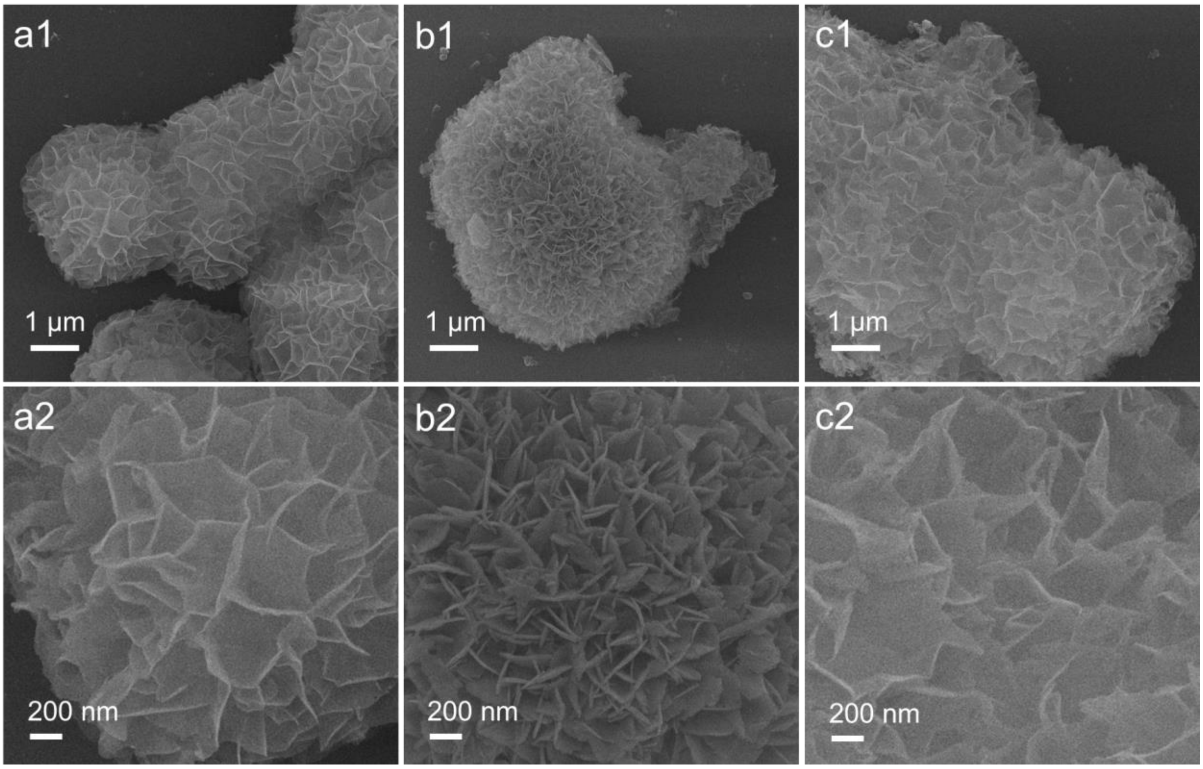

Figure S21 SEM images of a–c Ni/Al_2O_3, 0.5%Ir/Al_2O_3 and 0.5%$IrNi/Al_2O_3$ samples, respectively.

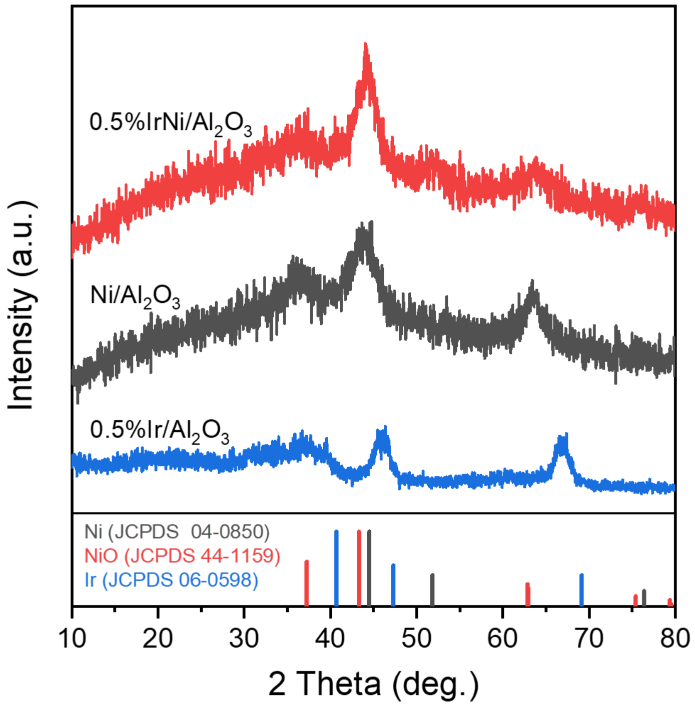

Figure S22 XRD patterns of pristine Ni, pristine Ir and Ir_1Ni SAA samples.

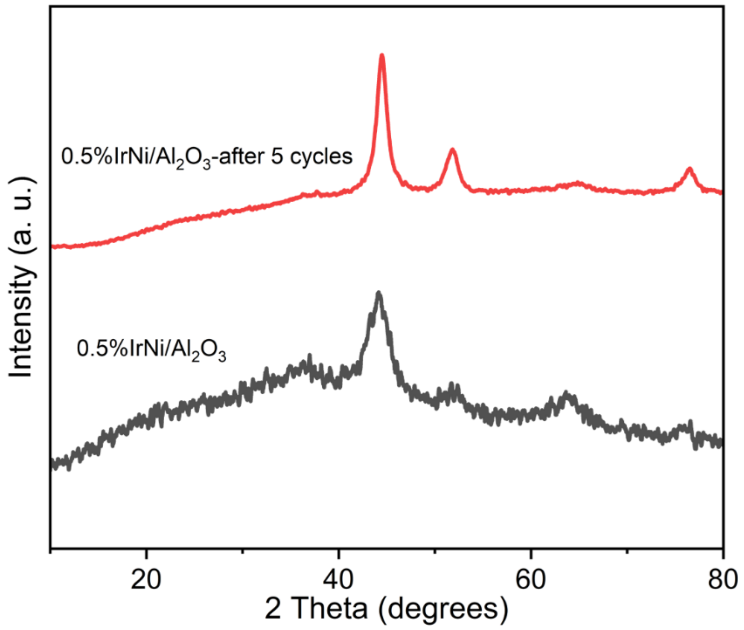

Figure S23 XRD pattern of the fresh and the used Ir_1Ni SAA catalyst after 5 cycle times.

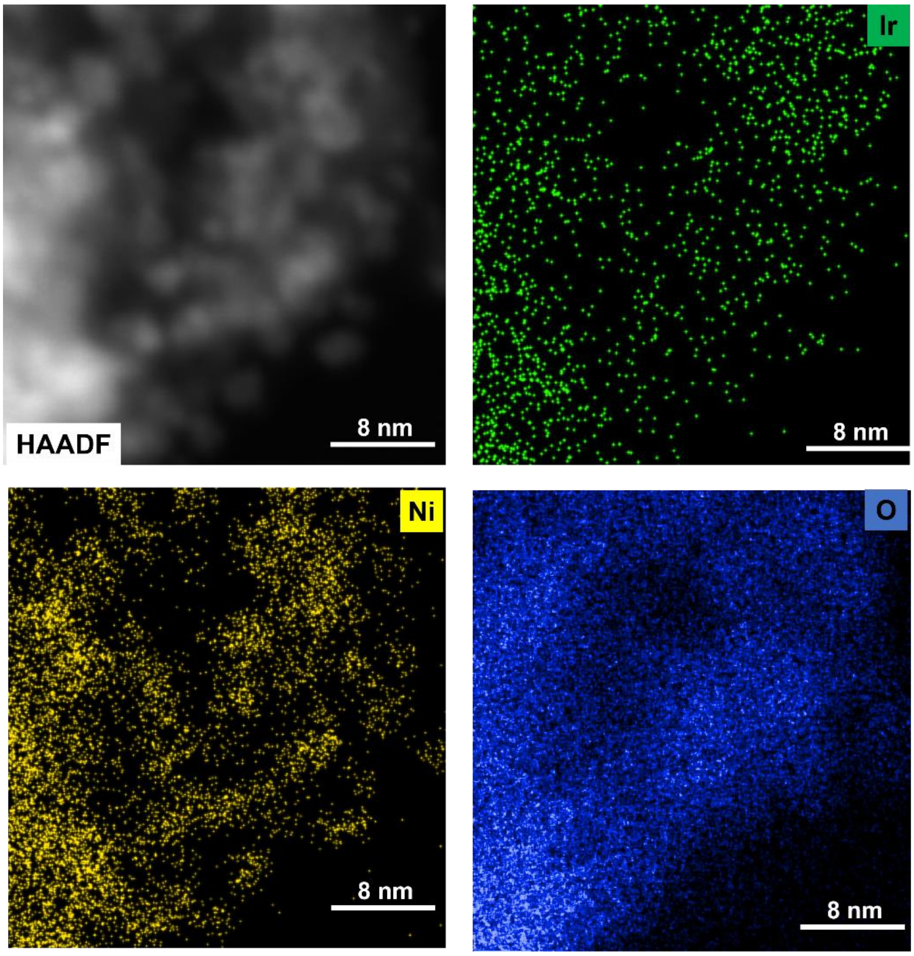

Figure S24 Element EDS mapping images of used Ir_1Ni SAA sample.

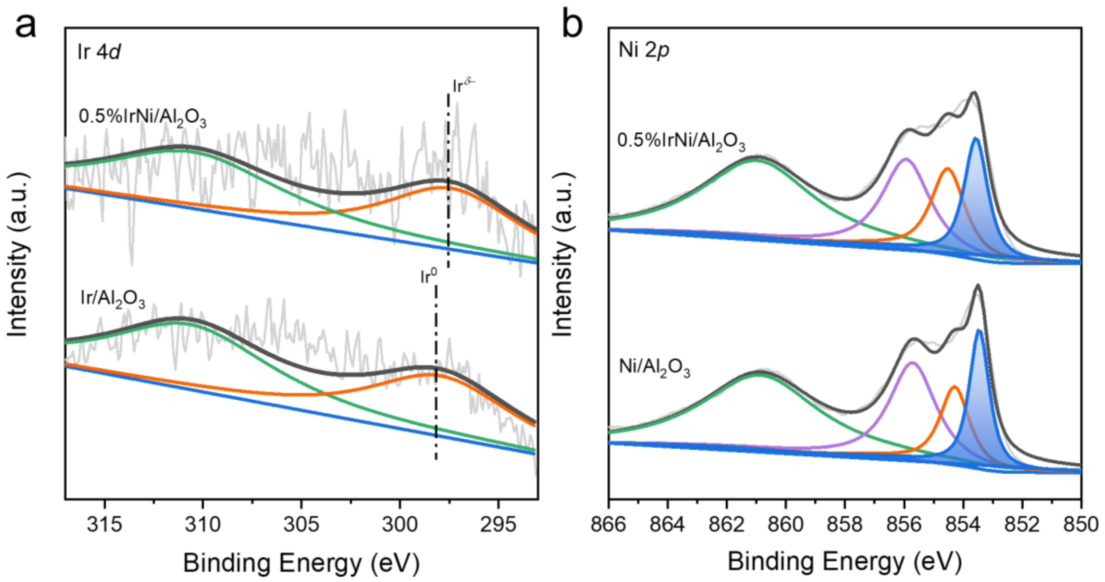

Figure S25 XPS spectra of Ir/Al_2O_3, Ni/Al_2O_3 and Ir_1Ni SAA sample.

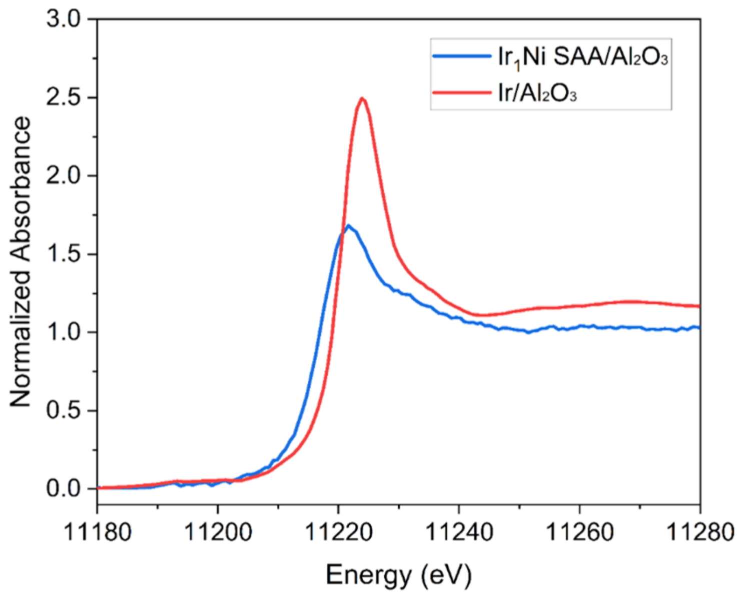

Figure S26 Ir K-edge XANES spectra of Ir_1Ni SAA/Al_2O_3 and Ir/Al_2O_3 samples.

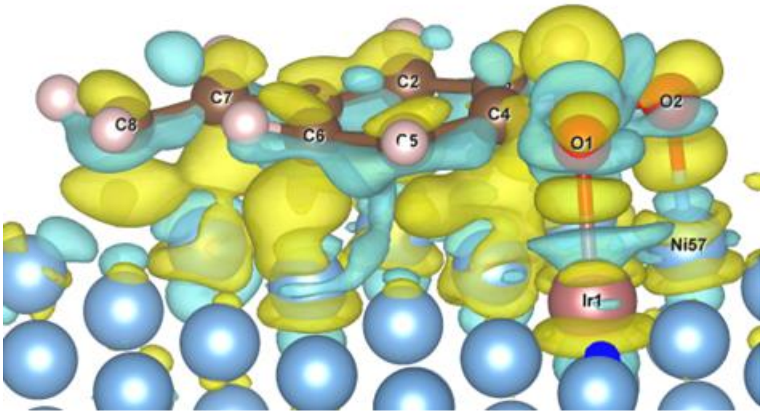

Figure S27 Charge difference distribution for 4-NS adsorption over $Ir_1Ni(111)$ surface (the cutoff of the density-difference isosurface is 0.003 e Å^{-3}).

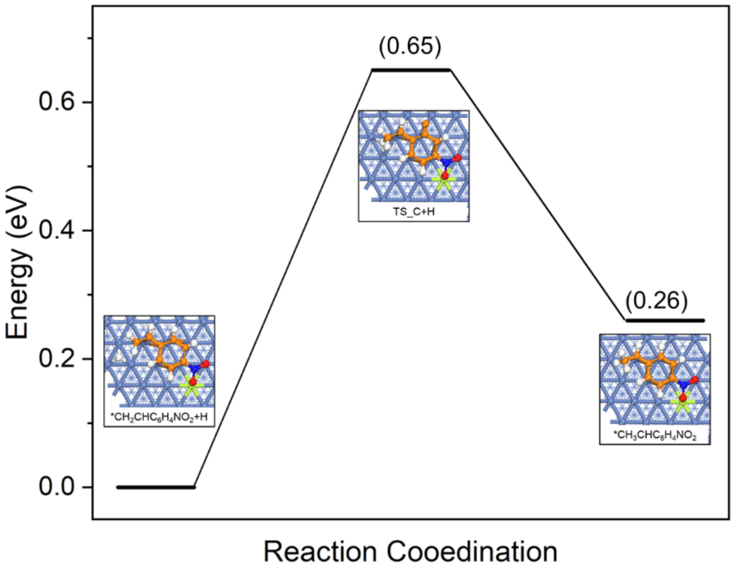

Figure S28 Potential energies profiles and corresponding optimized structures for C=C hydrogenation over $Ir_1Ni(111)$ surface.

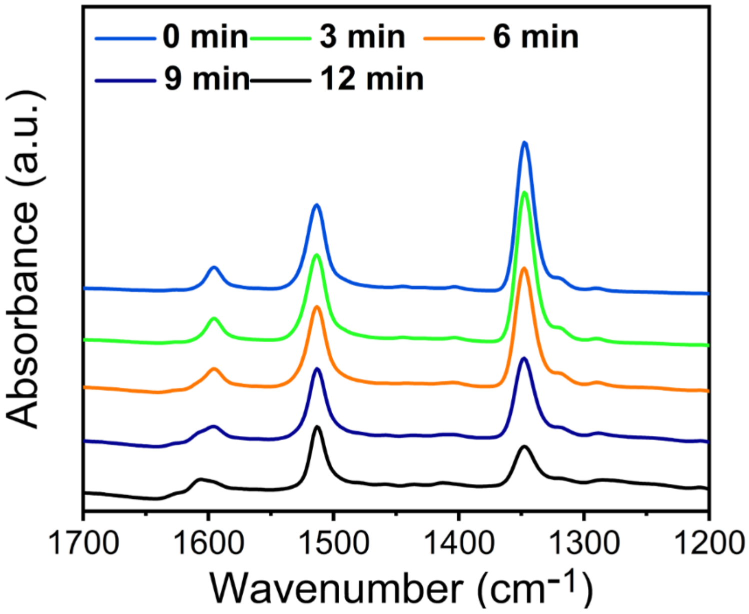

Figure S29 *In situ* FT-IR spectra of 4-NS hydrogenation in the presence of pure Ni catalyst.

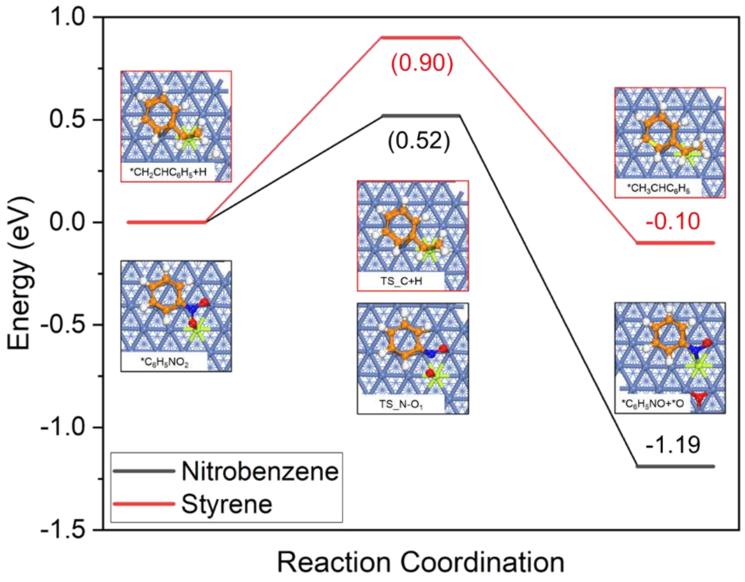

Figure S30 Potential energy profiles and corresponding optimized structures for C=C hydrogenation in styrene and N–O scission in nitrobenzene over Ir_1Ni SAA.

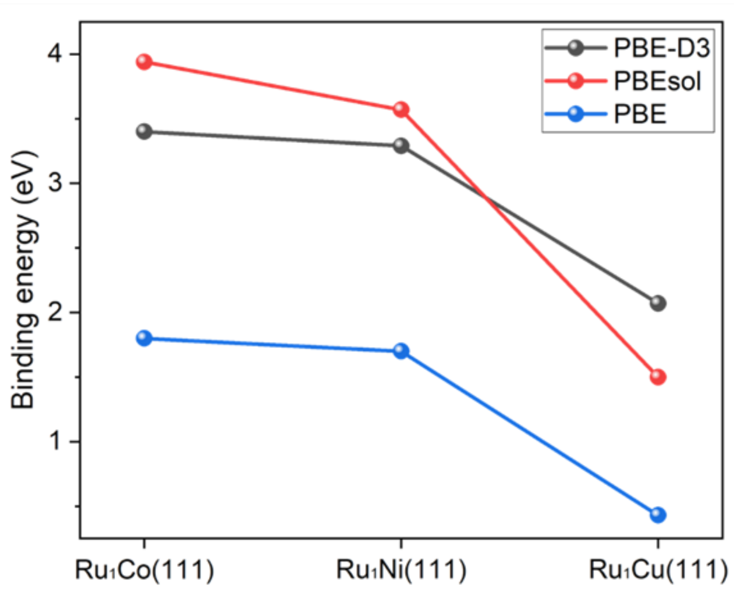

Figure S31 Binding energies of 4-nitrostyrene on $Ru_1Co(111)$, $Ru_1Ni(111)$ and $Ru_1Cu(111)$ as calculated with three functionals.

Table S1 Binding energy and N-O_1 bond length, M_1-O bond length.

**SAA**	**Co**	**Ru_1Co**	**Rh_1Co**	**Ir_1Co**	**Pd_1Co**	**Pt_1Co**
$d_{O\text{-}M}$/Å	2.020	2.104	2.177	2.158	2.320	2.323

$d_{N\text{-}O1}$/Å	1.323	1.329	1.306	1.326	1.287	1.294
E_{ads}/eV	3.36	3.40	3.22	3.18	3.07	3.00
**SAA**	**Ni**	**Ru_1Ni**	**Rh_1Ni**	**Ir_1Ni**	**Pd_1Ni**	**Pt_1Ni**
$d_{O\text{-}M}$/Å	2.016	2.104	2.197	2.129	2.303	2.333
$d_{N\text{-}O1}$/Å	1.319	1.333	1.309	1.346	1.291	1.293
E_{ads}/eV	3.14	3.29	3.16	2.97	2.89	2.98
**SAA**	**Cu**	**Ru_1Cu**	**Rh_1Cu**	**Ir_1Cu**	**Pd_1Cu**	**Pt_1Cu**
$d_{O\text{-}M}$/Å	2.114	2.052	2.174	2.168	2.346	2.397
$d_{N\text{-}O1}$/Å	1.309	1.335	1.302	1.316	1.286	1.288
E_{ads}/eV	1.80	2.07	1.89	1.88	1.70	1.66

Table S2 Total electronic population of atoms of gaseous 4-nitrostyrene molecule and adsorbed on the surface of Ir_1Ni SAA from Bader charge (e) analysis.

Atom	**O_1**	**O_2**	**N**	**C_1~C_6**	**C_7**	**C_8**	**H_1~H_7**
Gaseous 4-Nitrostyrene (e)	6.46	6.44	4.65	3.79~4.12	4.15	4.18	0.83~0.93
Adsorbed on Ir_1Ni SAA (e)	6.56	6.59	4.95	3.92~4.24	4.24	4.24	0.83~0.95

Table S3 Lattice parameters (in Å) of bulk Co, Ni and Cu as calculated with PBE-D3, PBEsol, PBE functionals and comparison to experiment values.

System	PBE-D3	PBEsol	PBE	Expt
Co	3.518	3.471	3.516	3.544[18]
Ni	3.524	3.471	3.516	3.524[19]
Cu	3.631	3.561	3.632	3.615[20]

References

1. Kresse, G.; Furthmüller, J. Efficiency of Ab-Initio total energy calculations for metals and semiconductors using a plane-wave basis set. *Comput. Mater. Sci.* **1996**, *6*, 15−50.

2. Kresse, G.; Furthmüller, J. Efficient iterative schemes for Ab-initio total-energy calculations using a plane-wave basis set. *Phys. Rev. B: Condens. Matter* **1996**, *54*, 11169−11186.

3. Blöchl, P. Projector augmented-wave method. *Phys. Rev. B: Condens. Matter* **1994**, *50*, 17953−17979.

4. Kresse, G.; Joubert, D. From ultrasoft pseudopotentials to the projector augmented-wave method. *Phys. Rev. B: Condens. Matter* **1999**, *59*, 1758−1775.

5. Perdew, J.; Burke, K.; Ernzerhof, M. Generalized Gradient Approximation Made Simple. *Phys. Rev. Lett.* **1996**, *77*, 3865–3868.

6. Grimme, S.; Antony, J.; Ehrlich, S.; Krieg, H. A Consistent and Accurate Ab Initio Parametrization of Density Functional Dispersion Correction (DFT-D) for the 94 Elements H-Pu. *J. Chem. Phys.* **2010**, *132*, 154104.

7. Perdew, J.; Ruzsinszky, A.; Csonka, G.; Vydrov, O.; Scuseria, G.; Constantin, L.; Zhou, X.; Burke, K. Restoring the density-gradient expansion for exchange in solids and surfaces. *Phys. Rev. Lett.* **2008**, *100*, 136406.

8. Henkelman, G.; Uberuaga, B.; Jónsson, H. A climbing image nudged elastic band method for finding saddle points and minimum energy paths. *J. Chem. Phys.* **2000**, *113*, 9901−9904.

9. Heyden, A.; Bell, A. T.; Keil, F. J. Efficient methods for finding transition states in chemical reactions: Comparison of improved dimer method and partitioned rational function optimization method. *J. Chem. Phys.* **2005**, *123*, 224101.

10. Hammer B, Nørskov J K, Electronic Factors Determining the Reactivity of Metal Surfaces. *Surf. Sci.* **1995**, *343*, 211−220.

11. Henkelman, G., Arnaldsson, A.; Jo´nsson, H. A fast and robust algorithm for Bader decomposition of charge density. Comput. Mater. Sci. **2006**, *36*, 354–360.

12. Sanville, E., Kenny, S. D., Smith, R.; Henkelman, G. Improved grid-based algorithm for Bader charge allocation. J. Comput. Chem. **2007**, *28*, 899–908.

13. Tang, W., Sanville, E.; Henkelman, G. A grid-based Bader analysis algorithm without lattice bias. J. Phys.: Condens. Matter. **2009**, *21*, 084204.

14. Momma, K.; Izumi, F. VESTA 3 for three-dimensional visualization of crystal, volumetric and morphology data. J. Appl. Crystallogr. **2011**, *44*, 1272–1276.

15. He, S.; Li, C.; Chen, H.; Su, D.; Zhang, B.; Cao, X.; Wang, B.; Wei, M.; Evans, D.; Duan, X. A surface defect-promoted Ni nanocatalyst with simultaneously enhanced activity and stability. *Chem. Mater.* **2013**, *25*, 1040–1046.

16. Ravel, B. & Newville, M. ATHENA, ARTEMIS, HEPHAESTUS: data analysis for X-ray absorption spectroscopy using IFEFFIT. *J. Synchrotron Radiat.* **2005**, *12*, 537–541.

17. Funke, H., Chukalina, M. & Scheinost, A. C. A new FEFF-based wavelet for EXAFS data analysis. *J. Synchrotron Radiat.* **2007**, ***14***, 426–432.

18. Yang, W.; Le, M.; Peng, S. Cobalt(II) and iron(II) complexes of 6-mercaptopurine. *Seances Acad.*

Sci. **1985**, *106*, 65−68.
19. Suh, I. K.; Ohta, H.; Waseda, Y. High-temperature thermal expansion of six metallic elements measured by dilatation method and X-ray diffraction. *J. Mater. Sci.* **23**, 757−760 (1988).
20. Straumanis, M. & Yu, L. Lattice parameters, densities, expansion coefficients and perfection of structure of Cu and of Cu–In α phase. *Acta Crystallogr.* **25**, 676–682 (1969).
